# miR-9 Acts as an OncomiR in Prostate Cancer through Multiple Pathways That Drive Tumour Progression and Metastasis

**DOI:** 10.1371/journal.pone.0159601

**Published:** 2016-07-22

**Authors:** S. J. Seashols-Williams, W. Budd, G. C. Clark, Q. Wu, R. Daniel, E. Dragoescu, Z. E. Zehner

**Affiliations:** 1 Department of Biochemistry & Molecular Biology, Virginia Commonwealth University, Richmond, Virginia, United States of America; 2 Department of Bioinformatics, Virginia Commonwealth University, Richmond, Virginia, United States of America; 3 Department of Chemistry, Virginia Commonwealth University, Richmond, Virginia, United States of America; 4 Department of Pathology, Virginia Commonwealth University, Richmond, Virginia, United States of America; Florida International University, UNITED STATES

## Abstract

Identification of dysregulated microRNAs (miRNAs) in prostate cancer is critical not only for diagnosis, but also differentiation between the aggressive and indolent forms of the disease. miR-9 was identified as an oncomiR through both miRNA panel RT-qPCR as well as high-throughput sequencing analysis of the human P69 prostate cell line as compared to its highly tumorigenic and metastatic subline M12, and found to be consistently upregulated in other prostate cell lines including DU-145 and PC3. While miR-9 has been characterized as dysregulated either as an oncomiR or tumour suppressor in a variety of other cancers including breast, ovarian, and nasopharyngeal carcinomas, it has not been previously evaluated and proven as an oncomiR in prostate cancer. miR-9 was confirmed an oncomiR when found to be overexpressed in tumour tissue as compared to adjacent benign glandular epithelium through laser-capture microdissection of radical prostatectomy biopsies. Inhibition of miR-9 resulted in reduced migratory and invasive potential of the M12 cell line, and reduced tumour growth and metastases in male athymic nude mice. Analysis showed that miR-9 targets e-cadherin and suppressor of cytokine signalling 5 (SOCS5), but not NF-ĸB mRNA. Expression of these proteins was shown to be affected by modulation in expression of miR-9.

## Introduction

Prostate cancer (CaP) is the most common cancer for men in the United States other than skin cancer, and the second leading cause of cancer deaths in the US, with over 29,000 fatalities each year [1]. The current standard for diagnosis of a potential prostate cancer is a rise in prostate-specific antigen (PSA) levels as a screening test, followed by manual examination, and ultrasound-guided transrectal biopsy [2]. Interventions tend to have drastic consequences for the diagnosed male; radical prostatectomies, cryosurgery, and androgen ablation therapy significantly affect patient quality of life through high levels of incontinence, psychological, and sexual side effects [3]. Thus, the identification of new screening practices is critical to diagnosing not only prostate cancer, but also differentiating between the aggressive and indolent forms of the disease.

microRNAs are a class of small RNAs that were described in *C*. *Elegans* in 1993 [4], and have since emerged as major regulators of protein levels through attenuation of translation at the ribosome [5–8]. microRNAs, or miRs, are 19–22 nucleotide single-stranded RNA sequences that are guided by a protein complex to their mRNA targets, typically in the 3’-UTR of the mRNA. microRNA transcription is often driven by standard transcription factor activation, including c-Myc and NF-kB [9–11], and most miRs are transcribed by RNA Polymerase II [12]. microRNAs can bind either perfectly or imperfectly to an mRNA target, thus making it possible for one miR to attenuate the translation of tens to hundreds of different targets. Additionally, miRs have been shown to impact all aspects of the proteome, from cell proliferation and apoptosis to mitochondrial and metabolic processes, to cytoskeleton and secreted products [8,13]. Thus, microRNAs have recently been subject to intense scrutiny as modulators of protein levels in cancer, as they are increasingly being shown to influence carcinogenesis and tumour progression.

The previously described progression cell lines P69 and M12 are a unique model for prostate cancer, in that P69, having been immortalized from a human non-neoplastic prostate epithelium section [14,15], is poorly tumorigenic and non-metastatic in nature. Having originated from a basal cell lineage, P69 and its sublines do not express the androgen receptor, and are thus androgen independent [15]. In contrast to its poorly tumorigenic P69 parent line, the M12 cell line, which was derived from 3 sequential subcutaneous injections of P69 cells into male nude athymic mice, is highly tumorigenic and metastatic upon orthotopic injection. The M12 cell line was found to harbour a chromosome 16:19 translocation resulting in the loss of one copy of chromosome 19, the restoration of which resulted in the F6 variant, which is mildly tumorigenic and not metastatic [16]. Thus, it is proposed that this set of cell lines can serve as amodel for prostate cancer research, in that the M12 and F6 variant are derived from P69 cells, and therefore share a common basic genetic complement which has developed a tumorigenic/metastatic phenotype mimicking what happens during tumour progression in man.

While prostate cell lines can act as good models for prostate cancer, any one cell line alone should not be used as a discovery tool for novel modulated species, including microRNAs. Moreover, a balanced discovery should include not only the easily obtainable cell lines, but also patient samples. In this way, confirmation of the preliminary results obtained from cell-line analysis can be confirmed as also occurring in the prostate cancer patient, and thus will not only be more relevant in identifying new biomarkers, but also in developing new therapeutics against prostate cancer. In this study, we proposed to identify microRNAs that have been modulated in prostate cancer through a progressive, sequential analysis that begins at the global miRNA level through both high-throughput sequencing (HTS) and RT-qPCR analysis, and proceeds through single-miR confirmatory analysis followed by evaluation of miR expression in multiple additional prostate cancer cell lines. Finally, the modulated microRNA is validated in human patient samples through laser capture microdissection (LCM) of benign and tumor tissue, and consequence of modulating microRNA expression on cell behaviour is analysed in vitro and on tumor growth in vivo, as well as the analysis of relevant mRNA targets for microRNA binding by in vitro and in vivo experiments.

## Materials and Methods

### Cell Culture

The establishment, maintenance, and characterization of the SV40 T antigen-immortalized human prostate epithelial cell P69 and its sublines, M2182, M12, and F6 have been described previously [15–17]. Briefly, two types of tumors were formed from the subcutaneous injection of the P69 glandular epithelial cell line into 18 male, athymic nude mice and designated as lineage I and lineage II [15]. The M2182 cell line is from Lineage I, derived from an early passage (T_5_-T_19_) of the P69 cell line soon after immortalization. Subsequent analysis showed it was non-metastatic and weakly tumorigenic. The M12 subline was also derived from Lineage I cells, but following three sequential cycles of subcutaneous growth in male, athymic nude mice. Upon intra-prostate injection the M12 variant was shown to readily metastasize to lungs or diaphragm (5/5 mice) and was verified to have lost one copy of chromosome 19. Restoration of the second copy of chromosome 19 resulted in the F6 variant, which exhibited a notable decline in tumorigenicity and the loss of metastatic capability [16]. These cell lines, as well as DU-145, were generous gifts from Dr. Joy Ware, Virginia Commonwealth University, Richmond, VA. The PC-3 cell line was a gift from Dr. Zheng Fu, Virginia Commonwealth University. All cell lines were authenticated using STR analysis and were kept in culture for less than 2 months. PC-3 and DU-145 cell lines were maintained according to established protocols [18,19]. Stable transformations of an early passage of the M12 cell line with a vector containing a miR-9 inhibiting sequence or scrambled control (pEZX-AM03, Geneocopeia Inc.) were performed using TransIT^®^-LT1 Transfection Reagent (Mirus BIO LLC) according to the manufacturer’s instructions.

### Locked-Nucleic Acid miR Panel Analysis

RNA extracted from M12 and P69 cell pellets was subjected to reverse transcription for analysis on Exiqon miRCURY LNA^™^ Universal RT microRNA PCR Panels I and II (Verson 2.M) (Exiqon A/S) and qPCR was conducted in an Applied Biosystems 7900HT real-time PCR instrument (Life Technologies) according to the manufacturer’s instructions. Threshold and baseline settings were set according to protocol recommendations. The data was corrected for interplate variability using on-plate calibrators, and normalized against the global mean using Exiqon GenEx software. Expression changes were calculated in Microsoft Excel^®^ as using the 2^-Δ ΔC^_T_ method [20] as M12 expression relative to P69.

### High-Throughput Sequencing of Cell Lines

Small RNA was extracted from duplicate P69 samples, and single M2182, M12, and F6 samples using the miRVana^™^ RNA isolation method (Ambion-Life Technologies) according to the manufacturer’s specifications for the extraction of small RNA and eluted in 100 ul. Sample RNA Integrity and quantitation was analysed using the 2100 Bioanalyzer and Small RNA quantitation method (Agilent Technologies Inc) according to the manufacturer’s recommendations. RNA samples were sent to the Nucleic Acids Research Facility at Virginia Commonwealth University for paired end sequencing on the Illumina platform. Briefly, Small RNA library preparation was conducted using the NEBNext^®^ Multiplex Small RNA Library Prep Set for Illumina^®^ (Set 1) (New England Biolabs). High throughput sequencing was conducted using the HiSeq 2500 (1x150) (Illumina^®^). Adapter trimming and sequence analysis was conducted using Flow, v3.0 (Partek^®^ Incorporated, St. Louis, Missouri, USA) using Bowtie 2 (v2.1.0) and miRbase v20 for alignment and annotation. miR sequencing reads were normalized using the reads per million (RPM) formula: (read counts of an individual miRNA/sum of read counts of all mappable miRNAs) multiplied by 1x10^6^.

### Laser-Capture Microdissection

Frozen radical prostatectomy samples (n = 5) were obtained from the University Tissue and Data Acquisition and Analysis Core after informed written consent and following approved Virginia Commonwealth University Institutional Review Board (IRB) protocols (HM13417_CR1). Each sample was reviewed and scored by a board certified pathologist with expertise in prostate cancer diagnosis. Frozen tissue slices (8 μm) from the biopsy cores were placed on uncharged glass slides, and stained with hematoxylin and eosin (H&E) using a standard protocol. Laser capture microdissection (LCM) was performed using the Arcturus Veritas^™^ laser capture microdissection system (Life Technologies). Each tissue type (benign, tumour) was separately captured onto CapSure^®^ Macro LCM caps (Life Technologies). At least ten slides were captured for each patient included in the study. Total RNA was isolated from LCM caps using the ARCTURUS^®^ PicoPure^®^ RNA Isolation Kit (Life Technologies), following the manufacturers protocol. RNA quality and quantity were estimated using an Agilent RNA 6000 Pico chip with the Bioanalyzer 2100 (Agilent Technologies) using the manufacturer’s instructions.

### Constructs and transient transfections

A portion of the 3’-UTR of e-cadherin was previously cloned into pmiR-Report, the seed region subsequently mutagenized [10] and both wild type and mutated clones were obtained through Addgene (Plasmids 25038 and 25039; http://www.addgene.org). Transient co-transfections of the 3’-UTR fragments cloned into the luciferase reporter vector along with a renilla luciferase vector (Promega) for normalization were performed using TransIT^®^-LT1 Transfection Reagent (Mirus BIO LLC) according to the manufacturer’s instructions. Briefly, cells were plated such that they would reach approximately 50% density after 24 hours. The cells were then rinsed with 0.5 mL PBS, and serum-containing media replaced. The cloned 3’-UTR:vector (0.5 μg) was mixed with 50 μL of RPMI media and 1 μg of Renilla plasmid with no serum or additives and 1.5 μL of TransIT^®^-LT1 reagent at room temperature for 15 minutes. The solution was added dropwise to each well, and gently rocked back and forth. The transfections were incubated for 24 hours under standard cell culture conditions and lysed as described below.

A sixty nucleotide portion of the miR-9 binding site in the 3’-UTR of SOCS5 and the mutated analog were synthesized through Invitrogen for oriented cloning into pmiR-Glo (Promega). Mutations of the seed recognition site (S1 Table) were synthesized as indicated, and failure of the mutated target site to bind miR-9 was verified through RNAHybrid [21,22]. Transient transfections of the 3’-UTR dual reporter vectors were performed using TransIT^®^-LT1 Transfection Reagent using the plating conditions described above.

Luciferase assays were conducted using the Dual-Luciferase Reporter Assay System (Promega Corporation). 100 μL of 1X Passive Lysis Buffer (Promega Corporation) was pipetted into each well, and lysed according to the manufacturer’s instructions. Luciferase and Renilla measurements were taken in a GloMax^®^ 20/20 luminometer (Promega Corporation).

### RNA Isolation and quantitative real-time PCR

RNA was isolated using the miRVana^™^ RNA isolation method (Ambion-Life Technologies). Messenger RNA expression was determined using iScript^™^ cDNA Synthesis kit (Bio-Rad Laboratories), followed by qPCR reactions using 1X FastStart Universal SYBR Green Master Mix (Roche Diagnostics, Indianapolis, IN) and 10 μM primer pairs for the relevant mRNA target (GAPDH: Forward: 5’-ACCACAGTCCATGCCATCAC; Reverse: 5’-TCCACCACCCTGTTGCTGTA. E-Cadherin: Forward: 5’-GGTGCTCTTCCAGGAACCTC; Reverse: 5’-GAAACTCTCTCGGTCCAGCC. SOCS5: Forward: 5’-CCTCCTTCGGCCTTCACCTA; Reverse: 5’-TATAAAATCGTGACCAATAGCAGGC) with 3 μL of cDNA reaction and brought to 20 μL volume with nuclease-free water. qPCR was conducted in an Applied Biosystems 7300 real-time PCR instrument (Life Technologies) using standard conditions. Data was analyzed using SDS software v1.3.1 (Life Technologies), using automatic threshold and baseline settings. Each mRNA evaluated was analyzed in triplicate using a minimum of three separate cell passage RNA extractions. GAPDH was used as a normalization control, and relative expression was calculated using the comparative C_T_ method [20].

Expression of miRNA targets was determined using the miRCURY LNA^™^ Universal reverse transcription reaction according to the manufacturer’s instructions (Exiqon A/S). The cDNA was diluted and qPCR undertaken in an Applied Biosystems 7300 real-time PCR instrument (Life Technologies) using recommended cycling conditions. Data was analyzed using SDS software v1.3.1 (Life Technologies). Threshold and baseline settings were set according to protocol recommendations, with baseline correction between cycles 3 and 12 and an automatic threshold. miR-9 (Exiqon # 202240:MIMAT0000441) was analyzed against a cell line with a minimum of one biological and three technical replicates. SNORD48 (Exiqon # 203903) was used as a normalization control, and relative expression was calculated using the comparative C_T_ method [20].

### Western Blotting

Cultured cell pellets were lysed in 4% sodium dodecyl sulfate (SDS) in phosphate-buffered saline (PBS) with 1X PhosSTOP phosphatase inhibitor (Roche Diagnostics) and COMplete protease inhibitor (Roche Diagnostics), followed by sonication for five minutes at 4°C. Western blot analysis was performed using the following antibodies: anti-β-actin (C4) produced in mouse (Santa Cruz Biotechnologies), anti-e-cadherin (CDH1) produced in mouse (Sigma Aldrich), anti-NF-kB produced in rabbit (Santa Cruz), and anti-SOCS5 (M-300) produced in rabbit (Santa Cruz). β-actin was used as a loading control.

### Cell proliferation assay

The effect of miR-9 on cell growth was determined through proliferation assays. M12 and M12 cells transformed with the miR-9 inhibitor plasmid were plated (5 x 10^3^ cells) onto a 24-well plate. The adherent cells were released by incubating with 0.25% Trypsin-EDTA (Gibco-Life Technologies) after which the trypsin was inactivated by washing the cells in serum-containing media. The cells counted using a Coulter Counter^®^ Analyzer (Beckman Coulter, Inc) or Vi-Cell^™^ XR Cell Viability Analyzer (Beckman Coulter, Inc) using a trypan blue solution. Cells were >90% live when analyzed in this fashion.

### Migration and Invasion Assays

For evaluation of migratory capabilities, 5 x 10^4^ M12 cells stably transformed with either a scrambled control or miR-9 inhibitor were added to a ThinCert^™^ TC membrane support insert (Greiner Bio-one BVBA/SPRL) placed in the well of a 24-well plate. For invasion assays, 30 μL of a 10% solution of Cultrex^®^ Basement Membrane Extract (Growth Factor Reduced), (R&D Systems^®^) in RPMI 1640 was pipetted onto the membrane support insert, and incubated at 37°C for 1 hour prior to adding 1.25 x 10^6^ cells to the upper chamber. For both assays, cells were plated in medium without serum, and medium supplemented with 5% FBS and 10 ng/mL EGF in the lower chamber of the well as chemoattractant. The unit was incubated for 24 hours. Cells that did not invade were removed by a cotton swab, and the cells on the lower surface of the membrane were fixed with 0.025% gluteraldehyde, followed by staining with 0.1% leucocrystal violet in 10% EtOH and PBS. The membranes were excised and mounted to a microscope slide, and cell counts were performed in 5 random fields for each replicate, averaged, and expressed as relative to the control. Data was the result of a minimum of 3 independent experiments, each performed in triplicate.

### *In-vivo* studies

All experiments were conducted in strict accordance with the recommendations in the Guide for the Care and Use of Laboratory Animals of the National Institutes of Health. The protocol was approved by the Institutional Animal Care and Use Committee of Virginia Commonwealth University (VCU) (Protocol Number: AM10131). All surgery was performed under ketamine and xylazine anesthesia, and Carprofen was administered daily for three days after surgery and subsequently as needed to minimize suffering. All animals were housed in an IACUC approved barrier controlled vivarium in enclosures containing a single animal, and the animals were housed in ventilated enclosures with HEPA filtration to prevent spread of infectious disease. The Vivarium was under administration of the VCU Department of Animal Resource (DAR) and access to the facility was strictly controlled. Animals were monitored daily by study personnel and DAR animal husbandry staff. Researchers ensured that each animal was provided with adequate food, water and clean enclosures. Enclosures were changed at least once per week and as needed for water spills, excess elimination etc. All animal areas were equipped with diurnal lighting, set for 12 hour on/off cycles. Five mice were assigned to each experimental group and were injected with 2.0 x10^6^ cells into the subcutaneous space on the back of each mouse. Following injection of cells into mice, they were monitored daily for signs of distress and discomfort using an IACUC approved moribundity scale (S4 Table). Mice with a score of >2 on the scale were euthanized using CO_2_ asphyxiation. No animals in the experimental group died before euthanization. One mouse in the experimental group was euthanized prior to the experimental endpoint. All other mice were euthanized at the end of the experiment.

To assess tumorigenicity, M12 and M12 cells transformed with the miR-9 inhibitor plasmid were subcutaneously (SC) injected into the dorsal flank of male athymic nude mice (2x10^6^ cells per mouse, five mice per treatment) (Harlan Laboratories, Inc., Indianapolis, IN). Tumour growth was monitored weekly using caliper measurement, and tumour volume calculated as length x width^2^/2 (mm^3^) over a period of 45 days until sacrifice. The tumors were excised following euthanization.

To assess metastatic potential, 3 male athymic nude mice were orthotopically injected with (10^6^ cells per mouse) of M12 or M12 cells transformed with the miR-9 inhibitor plasmid. The mice were sacrificed at 76 days, and peritoneal metastatic sites were counted.

### Data Analysis

Data are presented as the mean ± standard deviation. Statistical analyses were conducted in the Microsoft^®^ Excel software platform, using the independent Student's t-test, assuming equal variance between the two groups. Statistical significance was defined as p ≤ 0.05.

## Results

### High-Throughput Sequencing and Microarray Panel Analysis of M12 and P69 cell lines

Locked-nucleic acid (LNA) RT-qPCR panel analysis of the M12 versus the P69 cell line was the starting point for our search for microRNAs that are truly modulated, not only in cell-line models, but also in human tumours. A large number of microRNAs dysregulated between the P69 and metastatic M12 subline were identified through the miR panel analysis. Out of 736 microRNAs assayed using the RT-qPCR panel analysis of the M12 versus the P69 cell line, 231 were found to be OncomiRs (≥2 fold increase in M12 vs. P69 cell lines), 150 were found to be Tumour Suppressors (≤0.5 fold decrease in M12 vs. P69 cell lines), with the remaining microRNAs (355) within the normal range (S1 Fig). Even though the majority of microRNAs remained within the normal range, this still left many potential tumour suppressors and oncomiRs to evaluate (S2 Table). Because of the high numbers of dysregulated miRNAs identified, we chose to further narrow and verify candidate miRNAs through HTS analysis of the same cell lines. By HTS analysis, 161 dysregulated miRNAs were identified (S3 Table). Comparison of the two data sets resulted in a final list of candidate dysregulated miRs to be further evaluated.

The choice of which microRNAs to investigate further was accomplished through an evaluation of not only differences in the expression levels between P69 and M12 cell lines, but also literature reviews and analysis of potential targets using microRNA-target databases including TargetScan, DIANA, and miRDB [21–27]. Literature searches were conducted in order to determine whether the microRNAs in question had been previously identified as modulated in prostate or any other neoplasias, and single-miR analysis via RT-qPCR was conducted to confirm expression differences. Confirmatory RT-qPCR analysis of the selected miRs showed correlative results in fold expression differences from P69 to M12 for most, but not all, microRNAs selected.

**Fig 1:**
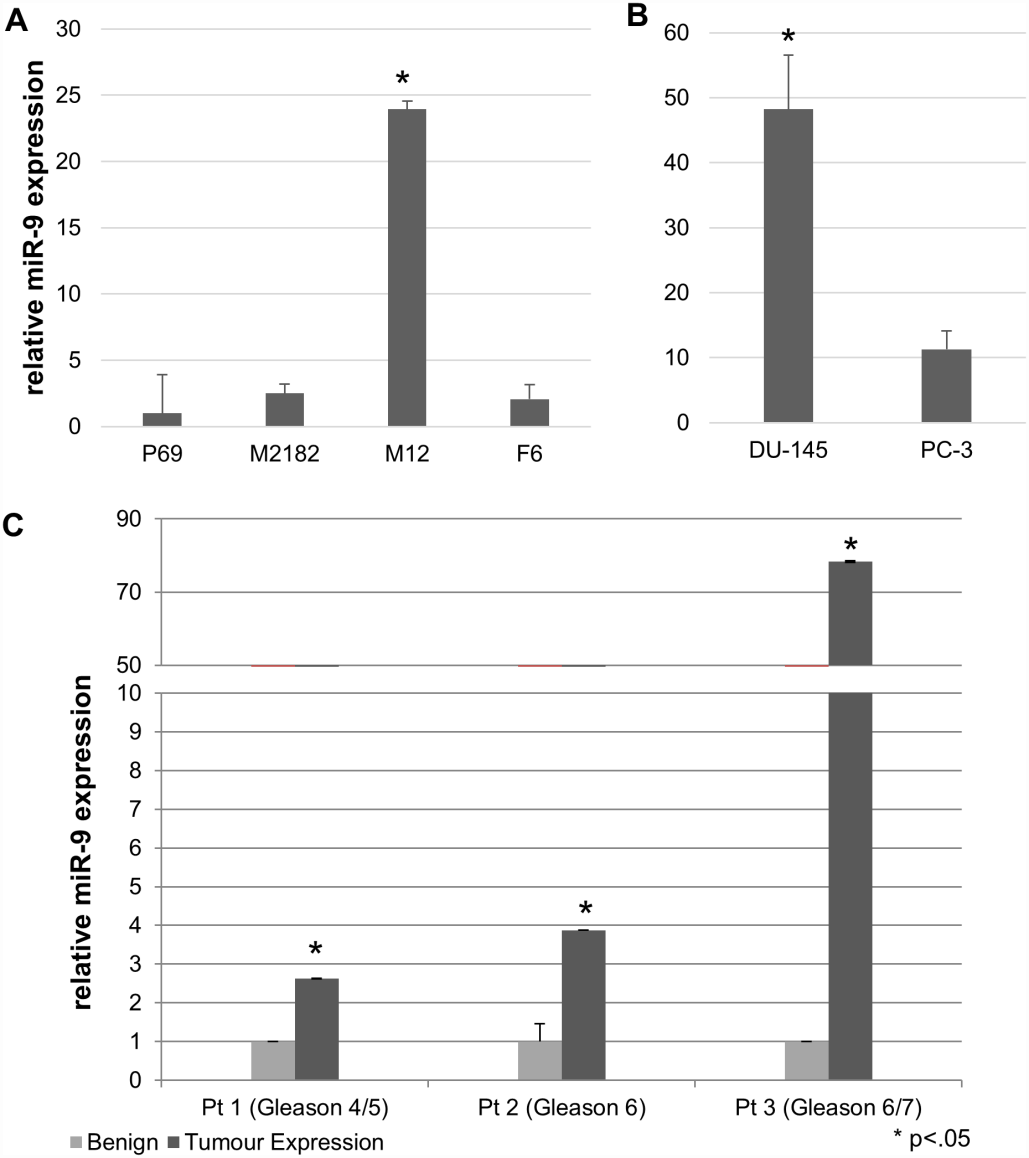
miR-9 levels are upregulated in all prostate cancer cell line models. A: miR-9 levels were significantly upregulated in M12 cells as relative to the parental P69 line (p = .002). Data is the mean of 3 independent experiments, each performed in duplicate (F6) or triplicate (P69, M2182, M12). B: miR-9 levels in DU-145 and PC3 cells are upregulated as relative to P69 cells (p <.001). Data was normalized to RNU48 (dC_T_) and expressed relative to P69 using the comparative C_T_ method. Data is the mean of three technical replicates. C: miR-9 expression is upregulated in 75% of positive patient tumours. miR-9 levels were measured in tumour and benign tissue separated from prostate biopsies using laser-captured microdissection (LCM). Data was normalized to RNU48 and expressed relative to the benign tissue using the comparative C_T_ method, and is the average of 3 technical replicates. miR-9 was undetected in both benign and tumour of the fifth patient, and thus was not included in the analysis.

miR-9 was chosen for further analysis, given its known role in other neoplasias and well-characterized proven targets [10,28–36]. A substantial increase in miR-9 levels was noted in the M12s versus the parental P69 cell line. Both HTS and panel analysis identified miR-9 as a potential oncomiR, with 3.017-fold (p = .000295) and 6.676-fold increases in M12 vs. P69, respectively (S2 and S3 Tables). This result was further verified through single-miR RT-qPCR analysis; miR-9 levels were high in M12, but not found to be significantly higher than the parental line when evaluated in the non-metastatic, poorly tumorigenic M2182 and F6 derivate (Fig 1a). RT-qPCR analysis of miR-9 against additional model cell lines showed significant upregulation in DU-145 and PC-3 cell lines in correlation to the observed upregulation in the M12 progression model (Fig 1b). LCM samples were captured from 5 patient biopsies at varying stages of prostate cancer classified as Gleason 4–7. RT-qPCR analysis showed that in the majority of patient samples (3 out of 4) miR-9 was elevated, but in patient 5 it was not detected in either benign or tumor tissue and thus could not be included in the analysis (Fig 1c). As expected, there is considerable variability in the nature of individual patient samples, most likely due to the heterogeneous nature of solid tumours, but miR-9 was elevated in 75% of the detected samples analyzed.

**Fig 2:**
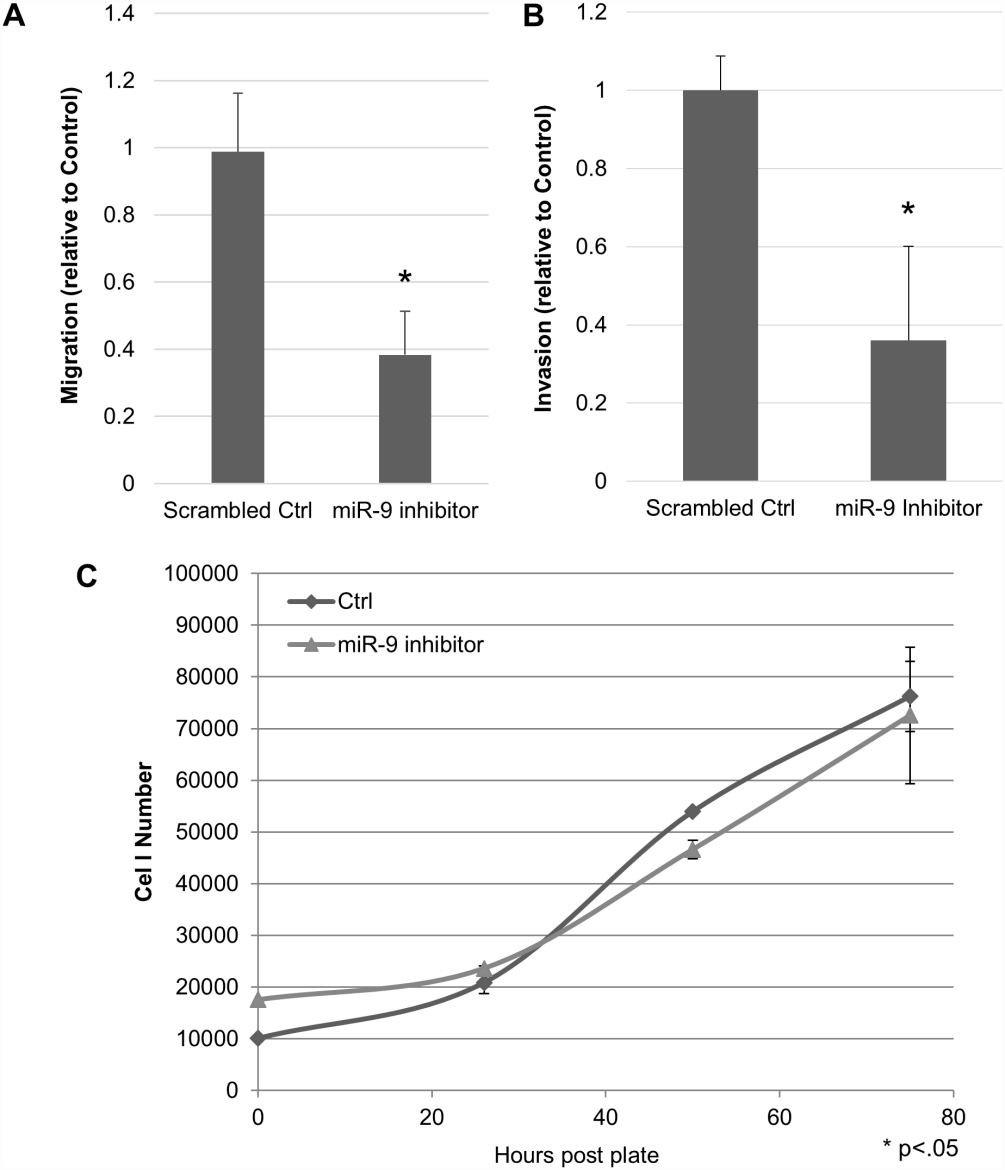
Inhibition of miR-9 significantly reduces migratory and invasive potential, but has no effect on cell proliferation. M12 cells were stably transformed with scrambled control or miR-9 inhibitor, plated on a ThinCert transwell membrane (with basement membrane added for invasion assay) and assessed for (A) migratory (p < 0.0001), (B) invasive potential (p < 0.0001), and (C) proliferation. Data is the mean of 3 independent experiments, each performed in triplicate (A&B) or one representative of three independent experiments (C).

Evaluation of miR-9’s effect on the tumorigenic potential of the M12 cells was performed through inhibition of miR-9 via stable transfection of a miR-9 sponge sequence. Inhibition of miR-9 had significant effects on migratory and invasive potential of the highly metastatic M12 cell line as compared to the scrambled control (p <0.0001 for both assays) (Fig 2a and 2b); however, miR-9 inhibition did not affect cell proliferation rates (Fig 2c). This is not surprising, given that the M12 cell line was immortalized using the SV40T antigen, which binds Rb, preventingthetumour suppressor protein from controlling cell cycle progression at the G1 checkpoint [14,37].

**Fig 3:**
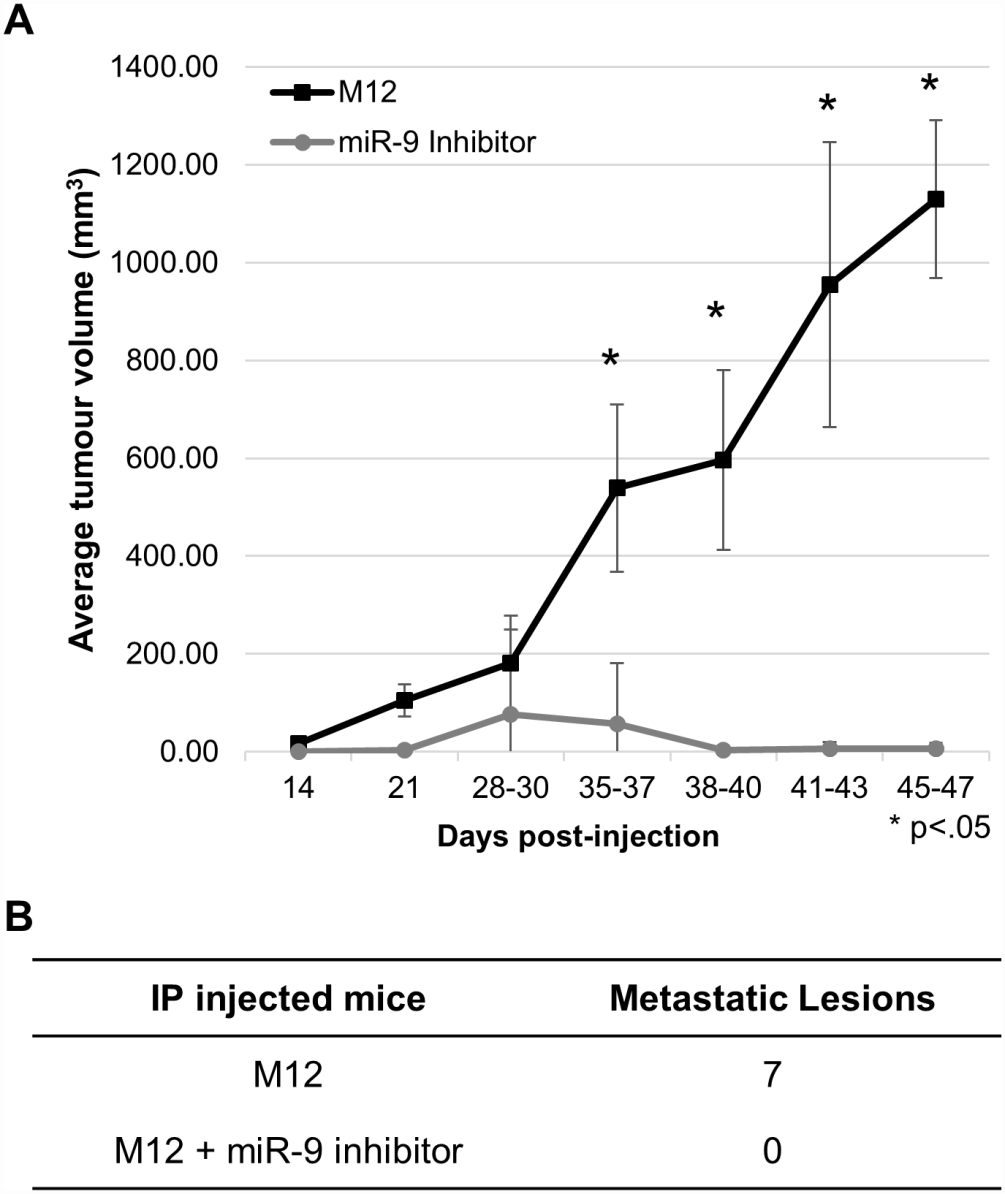
Tumour growth and metastasis is reduced upon miR-9 inhibition. A: Subcutaneous injections into the flank of the respective cell lines into nude athymic mice. Tumour reduction was significantly reduced in mice injected with M12 cells transformed with miR-9 inhibitor (p <.0001). Results are reported as the average ratio of tumour volume to the final average M12 tumour volume. N = 5 mice for each treatment. B: Intraprostatic (IP) injection into nude athymic mice resulted in 0 metastatic lesions when miR-9 was inhibited, as compared to 7 lesions in the mouse injected with M12 cells alone.

Subcutaneous injection of M12 cells stably transfected with miR-9 inhibitor into the flanks of male nude athymic mice showed a significantly lower level of tumour growth (p <0.0001) compared to mice injected with M12 cells (Fig 3a). Four of the five M12 injected mice developed tumours, but only two of the five M12+miR-9 inhibited mice developed tumours, which grew at a slower rate and size. Intraprostatic injection resulted in 7 metastatic sites for M12-injected mice, while neither of the M12+miR-9 inhibited mice had any observed metastatic sites by 76 days after injection (Fig 3b).

**Fig 4:**
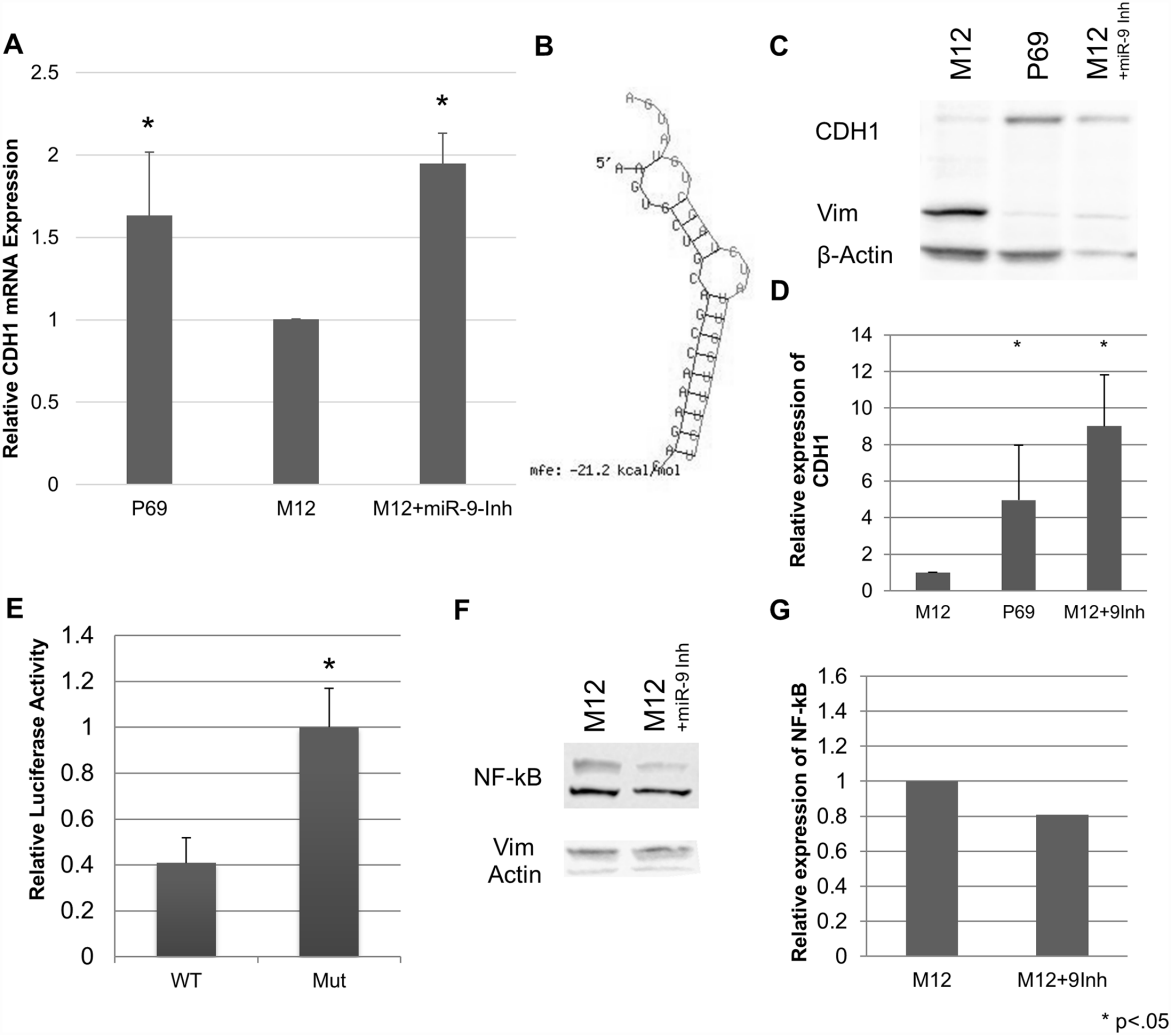
messenger RNA and protein levels of CDH1 are increased upon miR-9 inhibition. A: RT-qPCR analysis shows that mRNA levels are impacted by miR-9, and miR-9 inhibition relieves mRNA and protein levels (p<0.03 for P69 and miR-9 inhibited lines vs. M12). mRNA is normalized to GAPDH and reported as relative to the M12 cell line using the comparative C_T_ method. Results are compiled data from two biological replicates, each performed in triplicate. B: Proven binding site for miR-9 in e-cadherin. Adapted from RNAHybrid [21,22] analysis of CDH1 mRNA (NM_004360.3). miR-9 is the left sequence, and hybridized on the right is the complementary region of the 3’-UTR of CDH1. C: Western blot analysis and D: quantitation. Blot is representative of 5 independent experiments, Quantitation the average of 3 independent experiments normalized to β-Actin and reported as relative to the M12 cell line (p<0.05). (E) e-cadherin expression is suppressed by miR-9. M12 cells were transiently transfected with a firefly luciferase reporter construct containing a portion of the CDH1 3’-UTR, with the wild type or mutated miR-9 binding seed region along with a renilla luciferase plasmid. Firefly luciferase expression is reported as normalized to renilla luciferase activity and relative to mutated seed region expression. Results are the mean of 2 independent experiments, each performed in triplicate. (p <0.01). Western blot analysis shows that NF-kB levels do not change significantly between M12 or M12 cells stably transfected with miR-9 inhibitor. Blot (F) is representative of 3 independent experiments and quantitation (G) is from one representative experiment.

Given the strong evidence for miR-9’s contribution to oncogenic potential from these data, we felt confident that the miR has at least one significant target in prostate cancer that would prevent tumour progression when released from suppression. We therefore focused our efforts on evaluating miR-9’s proven targets in prostate cancer using the P69 and M12 progression model. As a known effector of the epithelial to mesenchymal transition (EMT), we were first interested in evaluating e-cadherin (CDH1), one of the proven targets for miR-9. In the early tumour cell’s transition to a motile phenotype, the change in expression from e-cadherin to vimentin is well known and described [10,38–40]. Previous work showed that vimentin is upregulated in the M12 cell line as compared to P69, and that vimentin upregulation is caused in part by loss of miR-17-3p in the M12 cell line [41]. Evaluation of e-cadherin mRNA levels was consistent with regulation by miR-9, exhibiting a reduced expression of mRNA levels in M12 compared to P69 (Fig 4a), which suggested cleavage activity upon miRNA binding to E-cadherin mRNA, even though miR-9 is not a perfect match to CDH1. Inhibition of miR-9 (M12+miR-9-Inh) resulted in rescue of e-cadherin mRNA. One miR-9 binding site in e-cadherin was identified through an RNAHybrid analysis of the entire mRNA (Fig 4B). This site, found in the 3’-UTR, has been shown previously to be directly bound by miR-9, and impacting its translation [10,38]. Interestingly, based on a TargetScan [25–27,42] analysis, miR-9 is the only conserved microRNA that binds to e-cadherin’s 3’-UTR. Western blot analysis confirmed that e-cadherin was highly expressed in P69 cells, but is almost completely lost in the M12 cells, with a corresponding change in vimentin protein levels, fitting with an EMT transition in M12 relative to the P69 cell line. Inhibition of miR-9 resulted in a corresponding rescue of CDH1 levels (Fig 4c), which upon correction for unequal loading by normalization to the internal control β-actin (Fig 4d), was considerably greater (~3-fold) than that detected in the parental P69 cell line. Transient transfections of luciferase constructs with the wild type or mutated target for seed recognition within a portion of CDH1's 3’-UTR verified that blockage of miR-9 binding resulted in loss of suppression as observed through a notable increase (~2.5-fold) in luciferase activity (Fig 4e).

NF-kB, another mRNA target known to be regulated by miR-9, is typically only shown as modulated when miR-9 suppression is lost, as is seen in carcinomas that exhibit a loss of miR-9 expression [28,31,33,43,44]. In these instances, relief of suppression of NF-kB results in initiation of transcription of a variety of pro-oncogenic and angiogenic genes. However, a concomitant decrease in NF-kB has not been observed in cancers where miR-9 is overexpressed. Similarly, no difference between NF-kB levels was observed between the M12 or M12+miR-9 inhibited cells (Fig 4f and 4g). Thus, control of NF-kB in prostate cancer cells may not be a relevant target for miR-9 regulation in prostate cancer as characterized by these model cell lines.

**Fig 5:**
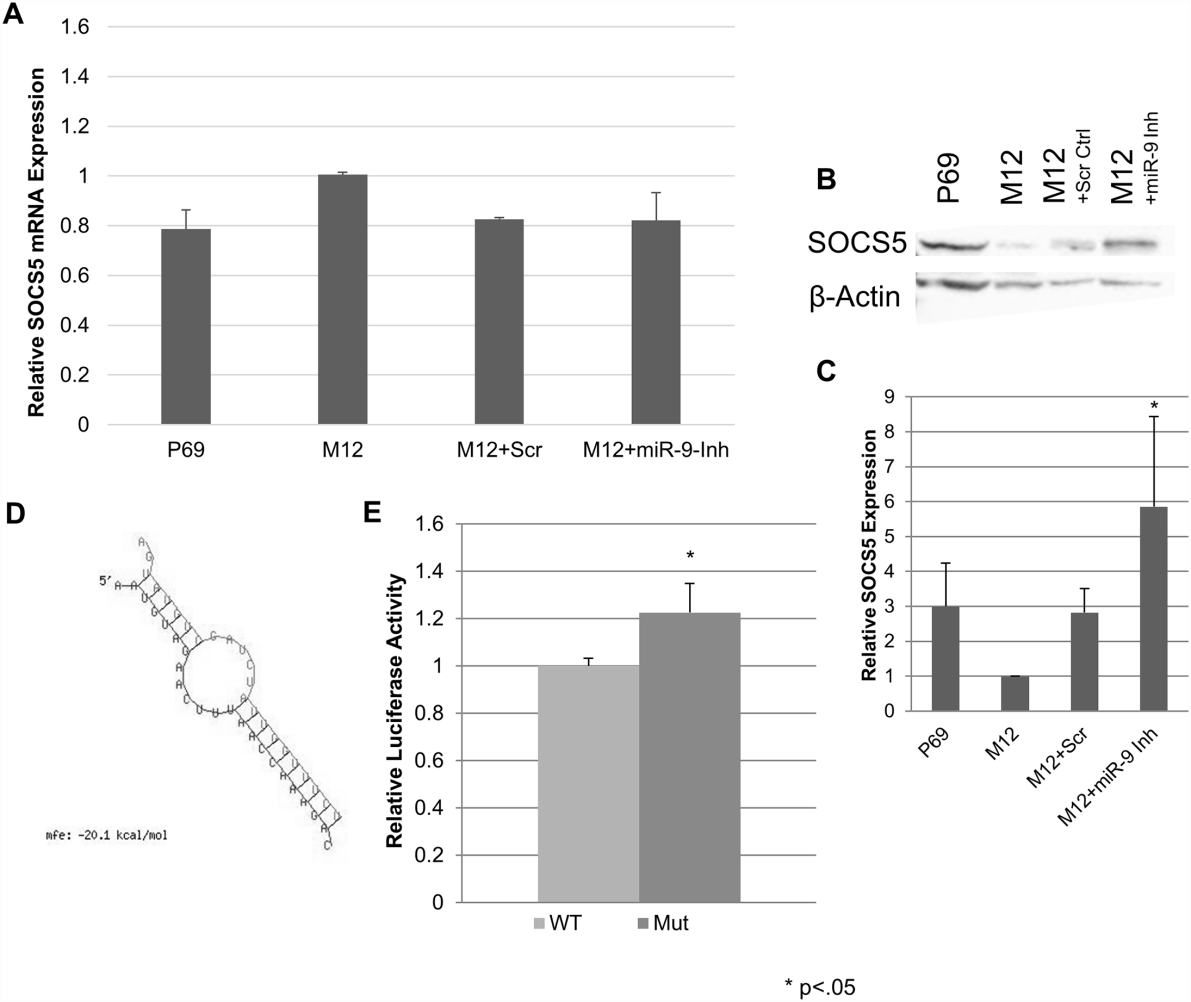
While protein levels of SOCS5 are increased upon miR-9 inhibition, messenger RNA levels are not significantly different. SOCS5 expression in the P69, M12, and M12 stably transformed with vector expressing miR-9 inhibitor. A: messenger RNA relative quantitation shows that mRNA levels are not significantly impacted by miR-9. mRNA is normalized to GAPDH and reported as relative to the M12 cell line; results are compiled data from three independent lysates, each performed in triplicate. Regardless of similar SOCS5 mRNA levels, (B) Western blot analysis and (C) quantitation show that miR-9 inhibition results in increased levels of SOCS5. Blot is representative of 6 independent experiments, Quantitation the average of 3 independent experiments normalized to β-Actin and reported as relative to the M12 cell line (p<0.05). (D) The proven SOCS5 mRNA:miR-9 hybrid [21,22]. miR-9 is the top sequence, and hybridized below is the complementary region of the 3’-UTR of SOCS5. (E) SOCS5 expression is suppressed by miR-9. M12 cells were transiently transfected with a dual luciferase reporter construct containing a portion of the SOCS5 3’-UTR, with the wild type or mutated miR-9 binding seed region. Firefly luciferase expression is reported as normalized to renilla luciferase activity and relative to mutated seed region expression. Results are the mean of 2 independent experiments, each performed in triplicate. (p = .016).

As a negative regulator of EGFR and JAK signaling pathway, the suppressor protein of cytokine signaling-5 (SOCS5) was recently identified as a direct target of miR-9 regulation [36]. Suppression of SOCS5 levels has been shown to attenuate signal transduction and ultimately transcription of a variety of pro-oncogenic, pro-angiogenic genes [45,46]. Evaluation of SOCS5 mRNA levels showed no significant differences between P69, M12, M12 cells stably transformed with a scrambled RNA sequence (M12+Scr) or stably transformed with the miR-9 inhibitor (M12+miR-9-Inh) (Fig 5a). However, western blot analysis indicated a strong difference in SOCS5 protein levels between the different cell types (Fig 5b and 5c). miRNA binding does not always result in a decrease of mRNA levels, as perfect seed matches to the miR and Argonaute 2 are required for message cleavage [47]. An evaluation of the previously identified SOCS5 mRNA target sequence in RNAHybrid against the miR-9 sequence showed binding with a large negative free energy value (Fig 5d), as well as high sequence conservation for the seed region binding across species (S2 Fig). Interestingly, although the matches between miR-9 and E-cadherin or SOCS5 mRNAs are not perfect, both exhibit a stable mfe structure, yet only CDH1 binding results in detectable cleavage of mRNA and not SOCS5, which adds to the complexity of what defines mRNA cleavage by the Argo/RISC complex. Transient transfections of luciferase constructs with the wild type or mutated target region within a portion of the SOCS5 3’-UTR showed that mutation resulted in the loss of suppression as observed through a small but statistically significant increase in luciferase activity (Fig 5E).

## Discussion

In this report, we identified microRNAs as oncomiRs or tumour suppressor miRs through a unique set of genetically related cell lines wherein the highly tumorigenic/metastatic cell line M12 is derived from the normal P69 glandular epithelial cell line through male athymic nude mice as a relevant model for tumor progression. Once identified through both high-throughput sequencing and RT-qPCR analysis, we sought to confirm miR-9 as an oncomiR through evaluation of other well-established prostate cancer cell lines followed by confirmation in human biopsy samples via laser-capture microdissection. Inhibition of miR-9 in M12 cells resulted in significantly reduced migratory and invasive potential in vitro, as well as reduced tumour growth in vivo. Moreover, no metastatic sites were observed in male, athymic nude mice injected orthotopically with M12+miR-9-Inh cells compared to injection with the parental M12 cell line. Evaluation of the expression signatures of known miR-9 targets indicated that while NF-kB is not modulated in this model, e-cadherin and SOCS5 are both affected by miR-9, resulting in increased levels when miR-9 is inhibited in M12 cells. Luciferase constructs of the e-cadherin and SOCS5 3’-UTRs showed that miR-9 suppression is effective in this prostate cancer model and is due to direct binding of miR-9 to mRNA target sequences.

### Identification of dysregulated miRNAs

RT-qPCR panel analyses of cell lines, while they should not be used as the sole method for identifying miRs that are modulated in neoplasias, can be an excellent launching point for identifying potential candidates, provided that the cell line model is accurate to *in-vivo* processes. In prostate cancer studies, various cell lines can serve different purposes. For example, the LNCaP series is an excellent model for identifying miRs that could contribute to the pathways required for androgen independence. However, since the LNCaP cell lines require coinjection with MS fibroblast cells for tumorigenesis and metastasis, they are not as good a model for initial tumour development. The P69/M12 progression model is a well characterized experimental system for studying initial aspects of human prostate cancer tumorigenesis [15,17,48], and thus we felt that a miR RT-qPCR panel comparing miR expression in these cell lines would best catalogue modulated miRs in a manner most faithful to human tumorigenesis. Variation between technical duplicates is well-known in miR RT-qPCR panel analysis; a study comparing data to single-miR analysis found anywhere from 0 to 10-fold differences in expression between the two platforms [49], similar to our observations. Thus, identified dysresgulated miRs were those that were consistently modulated (up or down) in duplicate panel analyses of both M12 and P69 cell lines.

To further narrow the field of candidates and gain additional confidence in the preliminary data, high-throughput small RNA sequencing of the same cell lines was performed. A smaller number of miRs were identified as dysregulated between the M12 and P69 cell lines using Illumina^®^ sequencing. This decrease in sensitivity was not unexpected and has been observed between the two platforms in other studies [50]. Using both sets of data, we could confidently narrow the list of candidate miRNAs for further analysis.

### miR-9

miR-9 was not a surprising find as dysregulated in prostate cancer. However, given its described role as oncomiR or tumour suppressor depending on the type of neoplasia [28–36], its validation as an oncomiR to drive prostate cancer progression has not been previously explored or proven. Here, miR-9 was overexpressed in all prostate cancer cell lines analyzed, and in 3 of the 4 patient LCM samples in which miR-9 was detected. Overexpression was also observed in canonical CaP cell lines DU145 and PC3, as well as M12 and patient samples (2.5–78 fold higher than benign). The dramatically higher miR-9 levels in DU145 as compared to that observed in PC3 and M12 cell lines correlate to a recent finding that overexpression of miR-9 and other miRNAs could be used as an indicator of neuroendocrine differentiation (NED) in CaP. Thus, high miR-9 levels could be expected to be observed in DU145, a prostate cell line derived from a brain tumour [19,51]. Besides this work and the report focusing on NED in CaP, there are only two other reports on miR-9 in prostate cancer, one being a microarray study and the others focused on the Androgen Receptor (AR). The microarray study found that miR-9 was consistently upregulated in prostate cancer, in tumour as compared to normal tissue. Additional upregulation has been observed in high-grade tumours as compared to lower-grade tumours [51, 52]; however, no specific work to validate the role of miR-9 as an oncomiR in prostate cancer progression was conducted in any of the three studies. miR-9 has many well-established protein targets, in particular, e-cadherin, NF-ĸB, SOCS5 and ETS-1. A modest but consistent decrease in the androgen receptor level has been shown when miR-9 was overexpressed in vitro [52], which could allow for an eventual switch from the early androgen-dependent tumour to a hormone-refractory state.

### E-Cadherin message and protein levels are suppressed by miR-9

Dysregulation of miR-9, although initially assumed as being overexpressed in carcinoma in general, has been shown through recent literature to be cancer type-specific; while in breast cancer, neuroblastoma and prostate cancer it is observed to be up-regulated [8,10,52], it is downregulated in metastatic melanomas. In these cell types, miR-9 suppresses expression of NF-ĸB, which results in increased levels of Snail1 and subsequent activation of e-cadherin [53]. Interestingly, higher levels of miR-9 have been associated with a better outcome in ovarian cancer, where it was shown to directly target BRCA1 [31].

The difference in miR-9 expression by cancer type could be due to promoter activity of miR-9, whether through PROX1, Snail1, or hypermethylation. miR-9 is also down-regulated in nasopharyngeal carcinoma (NPC), where it is implicated in the inflammatory response, specifically the Interferon-induced genes [54]. In this case, hypermethylation of the miR-9 promoter reduces transcription of the miRgene resulting in loss of suppression of CXCR, a chemokine receptor that has been shown to be expressed in a number of different types of tumors [30]. A second recent report showing the direct effect of miR-9 on CXCR identified that this loss of suppression resulted in accumulation of β-catenin through the Wnt pathway [40,55], and the subsequent transcription of JAK/STAT proliferative pathways [35].

**Fig 6:**
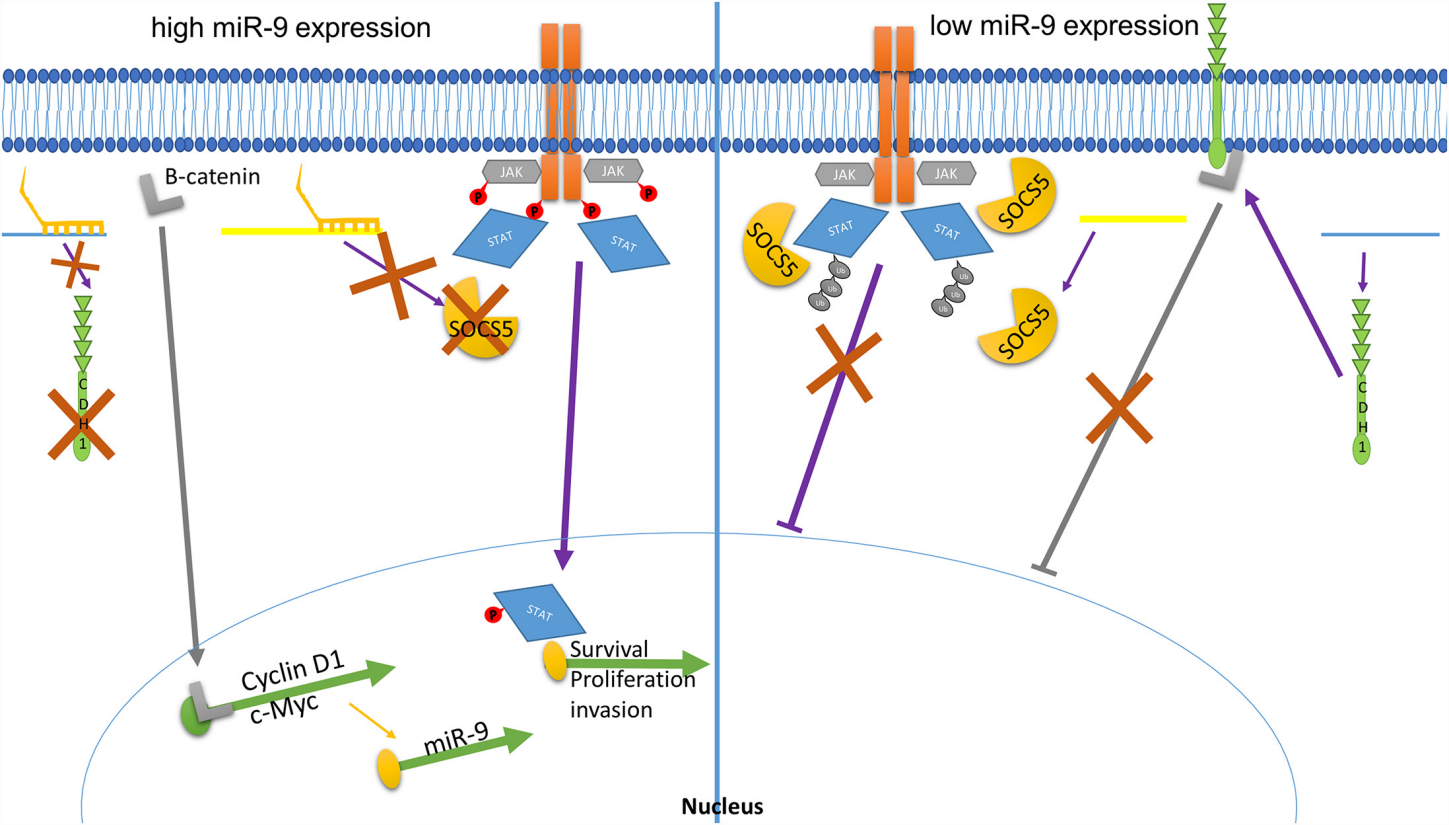
miR-9’s mode of action on tumour progression through e-cadherin and SOCS5. In the presence of miR-9 (left), e-cadherin message is cleaved and suppressed, resulting in less protein production. This allows for accumulation of β-catenin (grey “L”), which can then diffuse into the nucleus, activate transcription factors and drive pro-survival, pro-proliferation transcription including c-Myc and Cyclin D1. c-Myc then initiates additional miR-9 message through a positive feedback signal. SOCS5 translation is likewise suppressed when miR-9 is overexpressed, resulting in less protein production. This allows phosphorylation and signal transduction, resulting in p-STAT transcriptional activation of pro-survival, proliferation, and invasion/mestatasis oncogenes. In the noncancerous tissue in which miR-9 expression levels are low (right panel), e-cadherin is produced and sequesters β-catenin, preventing activation of transcriptional events. Likewise, SOCS5 is produced and works to prevent phosphorylation of JAK kinase and STAT while also promoting ubiquitination, thus attenuating the JAK/STAT signaling cascade, and resulting in decreased transcription of survival, proliferation, and invasion genes.

E-cadherin is known to sequester β-catenin, which also works to assist in dynamically connecting the adherens junctions to the cytoskeleton [38,40,56]. Loss of e-cadherin through miR-9 suppression would free β-catenin from the adherens complex, permitting movement into the nucleus and interaction with zinc finger transcriptional factors of the Tcf/Lef family to activate transcription of pro-metastatic, pro-angiogenic genes including VEGFA, Siamois, c-Myc and cyclin D1 [10,40] (Fig 6). Loss of e-cadherin has also been shown to reduce phosphorylation of β-catenin, which results in increased stability through lowered movement to the proteasome for degradation [38].

### SOCS5 protein levels are suppressed by miR-9

As a recently identified direct target for miR-9, loss of the tumour suppressor SOCS5 results in increased and prolonged activation of JAK/STAT pathways. The full function and target of RTK and JAK/STATS for SOCS5 is still unknown. However, it shares strong homology with the other members of the SOCS family, which are known to interfere with both JAK kinase and STAT protein activity, as well as promote their degradation through ubiquitination [46,57]. In the original report identifying miR-9 as a regulatory mechanism, SOCS5 expression was shown to be down-regulated when miR-9 was overexpressed, resulting in increased JAK1, STAT1 and STAT3 phosphorylation levels [36]. SOCS transcription is also known to be part of a negative feedback loop, in that STAT binding sites activate SOCS5 transcription for ultimate suppression of the signal transduction [46,57]. Even though the JAK/STAT pathway has been shown to be activated in M12 cells [14,15,58], the negative feedback loop that would ultimately attenuate JAK/STAT signaling is being prevented through suppression of SOCS5, as we have shown that SOCS5 protein levels in the M12 cells are lower than those observed in P69 (Fig 6).

## Conclusions

Analysis of a panel of over 700 miRs certainly generates a great deal of information, such that one research laboratory couldn’t begin to track down each of the modulated microRNAs. The majority of the miRs selected for further investigation are, for the most part, not well-described in prostate cancer, and the challenge will be to identify those mRNA targets that are suppressed through increased miR levels, or loss of microRNA expression leading to uncontrolled oncoprotein synthesis and the subsequent downstream effects.

The action of miR-9 is multifaceted; through suppression of miR-9, the e-cadherin that comprises cell-cell interactions characteristic of an epithelial lineage is suppressed. Thus, promotion from a stationary, embedded cell to a more motile phenotype is stimulated by the overexpression of miR-9, as is readily observed in the EMT transition in a variety of cancers. Loss of e-cadherin also results in release and stabilization of β-catenin, which can then move to the nucleus and activate transcription of VEGFA, c-Myc, Cyclin D1, and other pro-angiogenesis, proliferative genes. Given that c-Myc is a known activator of miR-9-3, this too represents a feedback loop that could further induce more miR-9 expression. Finally, action of miR-9 on SOCS5 results in loss of attenuation of the JAK/STAT pathways, which ultimately transduce growth signals to promote cell survival and proliferation. This is the first report to show a multi-targeted mode of action for miR-9 in a cancer; results observed in vivo indicate that both tumour growth and metastases are severely impacted by miR-9 inhibition. For those carcinomas in which miR-9 is overexpressed, inhibition of miR-9 could be a very effective therapeutic target, both in early neoplasias to prevent the EMT transition, but also in advanced, aggressive cancers to reduce proliferation and further metastasis.

## Supporting Information

S1 Fig Analysis of microRNA expression (fold difference in M12 vs. P69 cell lines). (PDF)

S2 Fig Targetscan analysis of miR-9 seed region binding site to SOCS5 3’-UTR (NM_014011.4) reveals high level of sequence conservation among species. (PDF)

S3 Fig Original western blots. (PDF)

S1 Table SOCS5 Mutations at the seed region sequences. (PDF)

S2 Table Tumour Suppressor (A) and OncomiR (B) microRNAs identified through miRcury RT-qPCR panel analysis of M12 and P69 cell lines. (PDF)

S3 Table miRNAs identified as significantly dysregulated through high-throughput sequencing of prostate cell lines (M12 vs. P69). (PDF)

S4 Table Moribundity scale for *in-vivo* experiments using mice. (PDF)
