# Supplementary material for: miR-9 Acts as an OncomiR in Prostate Cancer through Multiple Pathways That Drive Tumour Progression and Metastasis
Source: PLoS One. 2016 Jul 22;11(7):e0159601. doi: 10.1371/journal.pone.0159601 (PMC4957825; doi:10.1371/journal.pone.0159601)
Supplement: S1 Fig — (PDF) [file pone.0159601.s001.pdf]

miR-9 acts as an OncomiR in prostate cancer through multiple pathways that drive tumour progression and metastasis

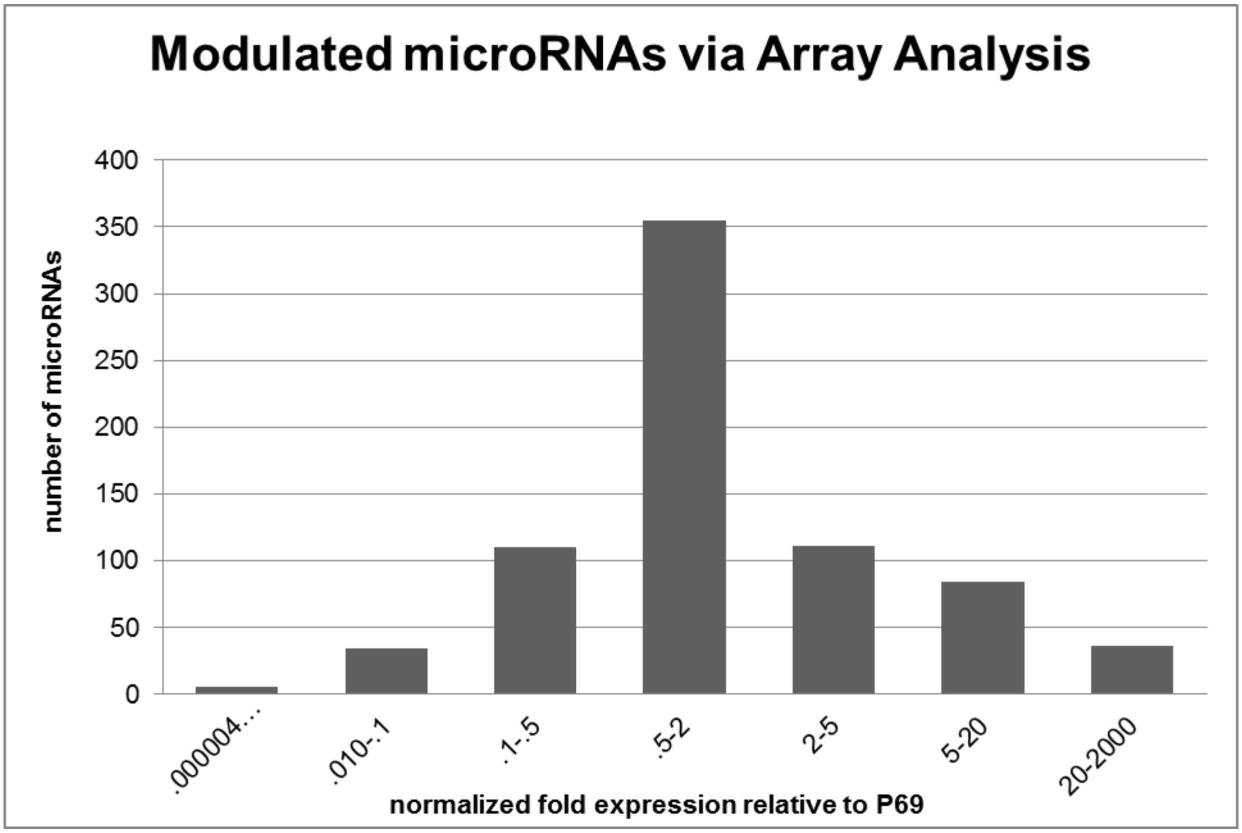

S1 Fig: Analysis of microRNA expression (fold difference in M12 vs. P69 cell lines) Cultured cells were pelleted, RNA isolated, and subjected to Exiqon miRCURY LNA™ microRNA system Human panels I and II (version 2.M). Panel I was performed in duplicate using 20 and 50 ng RNA; Panel II was performed once, using 50 ng RNA. Data was normalized using the global mean, and fold difference calculated. Positive values represent higher expression in M12 as compared to the P69 cell line.
