# Supplementary material for: miR-9 Acts as an OncomiR in Prostate Cancer through Multiple Pathways That Drive Tumour Progression and Metastasis
Source: PLoS One. 2016 Jul 22;11(7):e0159601. doi: 10.1371/journal.pone.0159601 (PMC4957825; doi:10.1371/journal.pone.0159601)
Supplement: S2 Fig — (PDF) [file pone.0159601.s002.pdf]

miR-9 acts as an OncomiR in prostate cancer through multiple pathways that drive tumour progression and metastasis

S2 Fig: Targetscan [26-28,44] analysis of miR-9 seed region binding site to SOCS5 3'-UTR (NM_014011.4) reveals high level of sequence conservation among species.

```
HSA  AAAUGUAG-AACUU--UAACCAAAGACU--UGUCCC
Ptr  AAAUGUAG-AACUU--UAACCAAAGACU--UGUCCC
Mml  AAAUGUAG-AACUU--UAACCAAAGACU--UGUCCA
Oga  AAAUGUAG-AACUU--UAACCAAAGACU--UGUCCA
Mmu  AGAUGCAG-ACCUU--UAACCAAAGACU--UGUUCA
Rno  AAAUGUGG-ACCUU--UAACCAAAGACU--UGUUCA
Cpo  AAAUGUAG-AACUUUGUAACCAAAGA-U--UAUCCA
Ocu  AAAUGUAG-AACUU--UAACCAAAGACU--UGUCCA
Sar  AAACGUAG-AACUU--UUACCAAAGACC--UGUCCA
Eeu  AAAUGAAG-AACUU--UAACCAAAGACU--UGUCCA
Cfa  AAAUGUAG-AAC-C--UAACCAAAGACU--UGUCCA
Fca  AAAUGUAG-AACUU--UAACCAAAGACU--UGUCCA
Eca  AAAUGUAG-AACUU--UAACCAAAGACU--UGUCCA
Bta  AAAUGUAGAAACUU--UAACCAAAGACU--UGUCCA
Dno  AAAUGUAG-AACUU--UAACCAAAGACU--UGUCCA
Laf  AAAUGUAG-AACUU--GAACCAAAGACU--UGUCCA
Ete  CAACGUAG-AACUU--UAACCAAAGACU--UGUCCG
Mdo  AAAUGUAG-AACUU--UUACCAAAGACU--UGUCCA
Gga  AAAUGUCC-UGUUU--UUACCAAAGAAUACUGUCAA
```
