# Supplementary material for: miR-9 Acts as an OncomiR in Prostate Cancer through Multiple Pathways That Drive Tumour Progression and Metastasis
Source: PLoS One. 2016 Jul 22;11(7):e0159601. doi: 10.1371/journal.pone.0159601 (PMC4957825; doi:10.1371/journal.pone.0159601)

S3 Fig: Original Western Blots

From Figure 4C (new = M12 + miR-9 Inhibitor new transfection analysis)

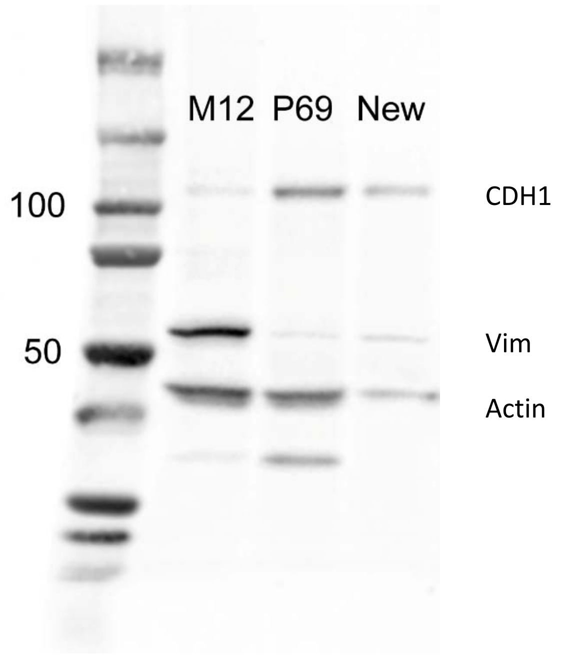

From Figure 4F: Two exposures of same blot.

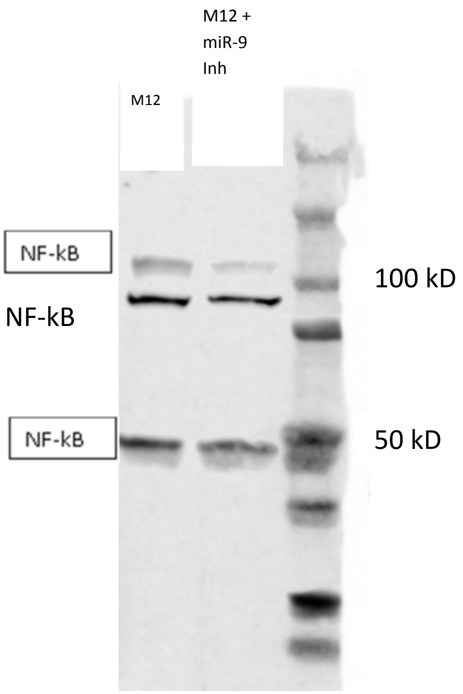

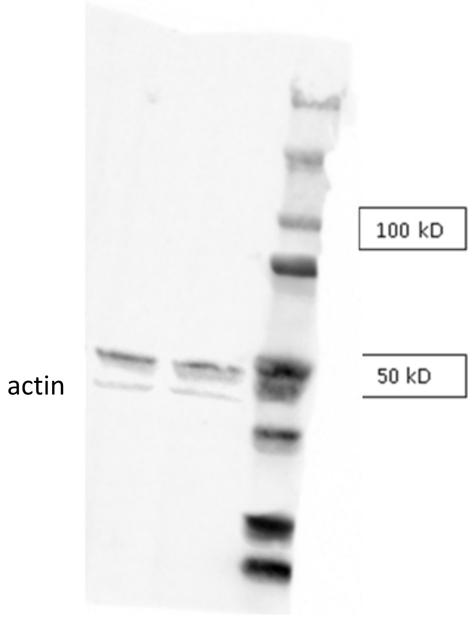

NF-kB exposure

Actin exposure

From Figure 5B

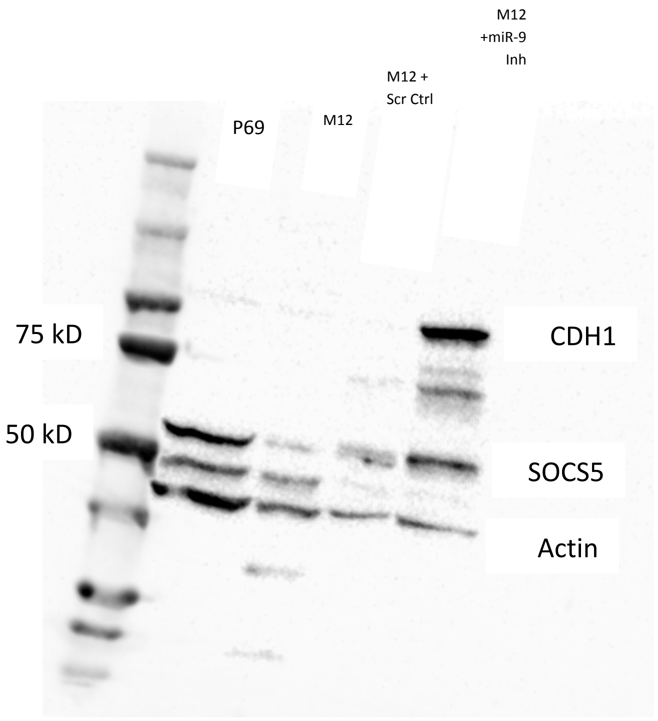

Supplement: S3 Fig — (PDF) [file pone.0159601.s003.pdf]
