# Supplementary material for: miR-9 Acts as an OncomiR in Prostate Cancer through Multiple Pathways That Drive Tumour Progression and Metastasis
Source: PLoS One. 2016 Jul 22;11(7):e0159601. doi: 10.1371/journal.pone.0159601 (PMC4957825; doi:10.1371/journal.pone.0159601)
Supplement: S1 Table — (PDF) [file pone.0159601.s004.pdf]

miR-9 acts as an OncomiR in prostate cancer through multiple pathways that drive tumour progression and metastasis

S1 Table: SOCS5 Mutations at the seed region sequences. Highlighted nucleotides indicates seed region, and red nucleotides in “MUT” were mutated from WT.

miR-9	AGUAUGUCGAUCUAUUGGUUUCU 5′
	||||||||||
SOCS5 WT	5′AUGUAGAACUUUAACCAAAGACUU
SOCS5 MUT	5′AUGUAGAACUUUAUGGUUUCUCUU
