# Supplementary material for: miR-9 Acts as an OncomiR in Prostate Cancer through Multiple Pathways That Drive Tumour Progression and Metastasis
Source: PLoS One. 2016 Jul 22;11(7):e0159601. doi: 10.1371/journal.pone.0159601 (PMC4957825; doi:10.1371/journal.pone.0159601)
Supplement: S2 Table — (PDF) [file pone.0159601.s005.pdf]

miR-9 acts as an OncomiR in prostate cancer through multiple pathways that drive tumour progression and metastasis

S2 Table A: Tumour Suppressor microRNAs identified through miRcury RT-qPCR panel analysis of M12 and P69 cell lines panels I and II (Version 2.M). Data is sorted by expression level, and values are fold expression difference in M12 from P69.

microRNA	Expression Difference	microRNA	Expression Difference	microRNA	Expression Difference
**hsa-miR-548m**	0.002	**hsa-miR-135b***	0.146	**hsa-miR-500**	0.319
**hsa-miR-127-3p**	0.002	**hsa-miR-132***	0.155	**hsa-miR-2113**	0.320
**hsa-miR-411**	0.005	**hsa-miR-520a-5p**	0.156	**hsa-miR-1227**	0.329
**hsa-miR-138-1***	0.005	**hsa-miR-29c***	0.158	**hsa-miR-425***	0.333
**hsa-miR-891b**	0.007	**hsa-miR-514**	0.160	**hsa-miR-146b-5p**	0.334
**hsa-miRPlus-C1089**	0.009	**hsa-miR-489**	0.161	**hsa-miR-140-3p**	0.338
**hsa-miR-1248**	0.017	**hsa-miR-145***	0.162	**hsa-miR-127-5p**	0.344
**hsa-miR-299-5p**	0.020	**hsa-miR-342-5p**	0.165	**hsa-miR-1181**	0.349
**hsa-miR-379**	0.020	**hsa-miR-23b***	0.169	**hsa-miR-587**	0.353
**hsa-miR-889**	0.025	**hsa-miR-556-3p**	0.173	**hsa-miR-379***	0.358
**hsa-miR-548a-5p**	0.028	**hsa-miR-23a***	0.178	**hsa-miR-1182**	0.359
**hsa-miR-323-3p**	0.031	**hsa-miR-1265**	0.187	**hsa-miR-876-3p**	0.365
**hsa-miRPlus-D1061**	0.036	**hsa-miR-148a***	0.189	**hsa-miR-296-3p**	0.370
**hsa-miR-34b***	0.037	**hsa-miR-143***	0.191	**hsa-let-7a-2***	0.371
**hsa-miR-487b**	0.039	**hsa-miR-1267**	0.193	**hsa-miR-603**	0.372
**hsa-miR-1238**	0.039	**hsa-miR-376a***	0.198	**hsa-miR-411***	0.374
**hsa-miR-409-3p**	0.042	**hsa-miR-105***	0.199	**hsa-miR-144**	0.374
**hsa-miR-135b**	0.043	**hsa-miR-1260**	0.210	**hsa-miR-1538**	0.378
**hsa-miR-614**	0.043	**hsa-miR-27a**	0.210	**hsa-miR-323-5p**	0.382
**hsa-miR-504**	0.044	**hsa-miR-125b-1***	0.212	**hsa-miR-758**	0.386
**hsa-miR-1914***	0.045	**hsa-miR-380**	0.212	**hsa-miR-770-5p**	0.392
**hsa-miR-520d-3p**	0.047	**hsa-miR-369-5p**	0.214	**hsa-miR-15b**	0.392
**hsa-miR-382**	0.048	**hsa-miR-572**	0.215	**hsa-miR-27b**	0.395
**hsa-miR-1185**	0.050	**hsa-miR-100**	0.222	**SNORD38B**	0.398
**hsa-miR-579**	0.051	**hsa-miR-1206**	0.225	**hsa-miR-1270**	0.400
**hsa-miR-659**	0.051	**hsa-miR-19b-2***	0.230	**hsa-miR-339-5p**	0.403

**hsa-miR-136**	0.055	**hsa-miR-138-2***	0.232	**hsa-miR-654-5p**	0.404
**hsa-miR-452***	0.057	**hsa-miR-598**	0.234	**hsa-miR-19b**	0.405
**hsa-miR-377**	0.058	**hsa-miR-376c**	0.235	**hsa-miR-630**	0.408
**hsa-miR-490-5p**	0.059	**hsa-miR-31**	0.237	**hsa-miR-449b***	0.413
**hsa-miR-936**	0.064	**hsa-miR-376b**	0.241	**hsa-miR-1256**	0.415
**hsa-miR-362-3p**	0.072	**hsa-miR-1258**	0.242	**hsa-miR-378***	0.416
**hsa-miR-616**	0.080	**hsa-miR-543**	0.256	**hsa-miR-212**	0.428
**hsa-miR-432**	0.085	**hsa-miR-191**	0.258	**hsa-miR-142-5p**	0.431
**hsa-miR-619**	0.086	**hsa-miR-432***	0.267	**hsa-miR-302d***	0.446
**hsa-miR-509-3p**	0.091	**hsa-miR-181a-2***	0.267	**hsa-miR-100***	0.448
**hsa-miR-1237**	0.095	**hsa-miR-302e**	0.267	**hsa-miR-555**	0.454
**hsa-miR-541***	0.096	**hsa-miR-448**	0.272	**hsa-miR-23b**	0.455
**hsa-miR-1179**	0.098	**hsa-miR-376a**	0.273	**hsa-miR-146a**	0.459
**hsa-miR-1236**	0.100	**hsa-miR-1245**	0.275	**hsa-miR-769-3p**	0.460
**hsa-miR-640**	0.105	**hsa-miR-493***	0.275	**hsa-miR-125a-3p**	0.469
**hsa-miR-135a**	0.122	**hsa-miR-649**	0.276	**hsa-miR-1253**	0.471
**hsa-miR-888***	0.124	**hsa-miR-924**	0.278	**hsa-miR-34c-5p**	0.472
**hsa-miR-342-3p**	0.127	**hsa-miR-191***	0.291	**hsa-miR-224***	0.475
**hsa-miR-588**	0.130	**hsa-miR-875-3p**	0.305	**hsa-miR-591**	0.479
**hsa-miR-527**	0.132	**hsa-miR-218-1***	0.306	**hsa-miR-450b-5p**	0.487
**hsa-miR-141***	0.135	**hsa-miR-526b***	0.306	**hsa-miR-636**	0.489
**hsa-miR-196b***	0.137	**hsa-miR-138**	0.310	**hsa-miR-720**	0.490
**hsa-miR-125b**	0.145	**hsa-miR-21**	0.312	**hsa-miR-1914**	0.492
**hsa-miR-516b***	0.146	**hsa-miR-1913**	0.316	**hsa-miR-203**	0.493

S2 Table B: OncomiR microRNAs identified through miRcury microarray analysis of M12 and P69 cell lines panels I and II (Version 2.M). Data is sorted by expression level, and values are fold expression difference in M12 from P69.

microRNA	Expression Difference	microRNA	Expression Difference	microRNA	Expression Difference
**hsa-miRPlus-A1027**	2.003	**hsa-miR-200c***	3.487	**hsa-miR-486-5p**	9.078
**hsa-miR-126**	2.004	**hsa-miR-188-5p**	3.557	**hsa-miR-570**	9.152
**hsa-miR-34c-3p**	2.006	**hsa-miR-642**	3.597	**hsa-miR-195***	9.307
**hsa-miR-515-3p**	2.007	**hsa-miR-326**	3.606	**hsa-miR-933**	9.341
**hsa-miR-621**	2.012	**hsa-miR-490-3p**	3.612	**hsa-miR-663b**	9.506
**hsa-miR-34a***	2.015	**hsa-miR-193b***	3.617	**hsa-miR-337-5p**	9.977
**hsa-miR-22**	2.026	**hsa-miR-450a**	3.620	**hsa-miR-551a**	10.377
**hsa-miR-365**	2.042	**hsa-miR-181c**	3.650	**hsa-miR-592**	10.486
**hsa-miR-491-5p**	2.071	**hsa-miR-208b**	3.741	**hsa-miR-513c**	10.656
**hsa-miR-96***	2.084	**hsa-miR-628-5p**	3.746	**hsa-miR-211**	10.954
**hsa-miR-1264**	2.092	**hsa-miR-154***	3.751	**hsa-miR-299-3p**	11.371
**hsa-miR-141**	2.093	**hsa-miR-7-1***	3.787	**hsa-miR-623**	11.895
**hsa-miR-589**	2.094	**hsa-miR-942**	3.830	**hsa-miR-508-3p**	12.437
**hsa-miR-26b**	2.105	**hsa-miR-187**	3.830	**hsa-miR-19b-1***	12.583
**hsa-miR-187***	2.157	**hsa-miR-135a***	3.855	**hsa-miR-517***	12.739
**hsa-miR-330-3p**	2.168	**hsa-miR-595**	3.858	**hsa-miR-767-3p**	12.925
**hsa-miR-32**	2.175	**hsa-miR-1244**	3.885	**hsa-miR-363***	13.280
**hsa-miR-885-3p**	2.211	**hsa-miR-558**	3.986	**hsa-miR-662**	13.298
**hsa-miR-346**	2.219	**hsa-miR-647**	4.044	**hsa-miR-520b**	13.474
**hsa-miR-28-3p**	2.227	**hsa-miR-943**	4.137	**hsa-miR-196a**	13.480
**hsa-miR-30a***	2.277	**hsa-miR-34a**	4.183	**hsa-miR-620**	13.515
**hsa-miR-668**	2.285	**hsa-let-7d**	4.191	**hsa-miR-518c***	13.620
**hsa-miR-628-3p**	2.295	**hsa-miR-429**	4.224	**hsa-miR-3180-3p**	13.753
**hsa-miR-1205**	2.307	**hsa-miR-675***	4.326	**hsa-miR-562**	15.051
**hsa-miR-1296**	2.322	**hsa-miR-548k**	4.433	**hsa-miR-767-5p**	15.266
**hsa-miR-30e**	2.327	**hsa-miR-370**	4.444	**hsa-miR-501-3p**	15.480
**hsa-miR-200b***	2.340	**hsa-miR-519e**	4.471	**hsa-miR-486-3p**	16.750
**hsa-miR-125b-2***	2.352	**hsa-miR-520f**	4.509	**hsa-miR-371-5p**	16.886
**hsa-miR-526b**	2.391	**hsa-miR-130a**	4.531	**hsa-miR-106a***	17.100
**hsa-miR-502-5p**	2.396	**hsa-miR-580**	4.630	**hsa-miR-338-5p**	17.177
**hsa-miR-137**	2.417	**hsa-miR-548i**	4.639	**hsa-miR-33b**	17.468
**hsa-miR-214**	2.435	**hsa-miR-890**	4.692	**hsa-miR-616***	17.628
**hsa-miR-223**	2.437	**hsa-miR-146a***	4.810	**hsa-miR-374b***	17.749
**hsa-miR-744**	2.443	**hsa-miR-583**	5.012	**hsa-miRPlus-A1031**	17.818
**hsa-miR-325**	2.483	**hsa-miR-188-3p**	5.021	**hsa-miR-1269**	17.957

**hsa-miR-7**	2.490	**hsa-miR-222***	5.023	**hsa-miR-372**	18.745
**hsa-miR-210**	2.497	**hsa-miR-518b**	5.143	**hsa-miR-557**	18.817
**hsa-miR-33b***	2.499	**hsa-miR-548o**	5.164	**hsa-miR-512-5p**	19.327
**hsa-miR-518e**	2.514	**hsa-miR-124**	5.174	**hsa-miR-202**	19.695
**hsa-miR-1249**	2.515	**hsa-miR-885-5p**	5.175	**hsa-miR-18b***	20.163
**hsa-miR-339-3p**	2.528	**hsa-miR-146b-3p**	5.355	**hsa-miRPlus-D1033**	21.296
**hsa-miR-760**	2.529	**hsa-miR-600**	5.403	**hsa-miR-675b**	21.361
**hsa-miR-30d**	2.555	**hsa-miR-597**	5.554	**hsa-miR-873**	21.467
**hsa-miR-1911***	2.561	**hsa-miR-15b***	5.574	**hsa-miR-1**	23.538
**hsa-miR-605**	2.583	**hsa-miR-381**	5.729	**hsa-miR-412**	23.833
**hsa-miR-148b**	2.601	**hsa-miR-99a**	5.742	**hsa-miR-491-3p**	24.610
**hsa-miR-877***	2.603	**hsa-miR-223***	5.753	**hsa-miR-498**	25.427
**hsa-miR-200a**	2.655	**hsa-miR-634**	5.801	**hsa-miR-937**	26.513
**hsa-miR-421**	2.664	**hsa-miR-518a-3p**	5.860	**hsa-miR-451**	27.084
**hsa-miR-499-5p**	2.731	**hsa-miR-596**	5.881	**hsa-miR-520d-5p**	27.591
**hsa-miR-1263**	2.741	**hsa-miR-30c-1***	5.921	**hsa-miR-556-5p**	27.696
**hsa-miR-26a-1***	2.751	**hsa-miR-516a-5p**	6.019	**hsa-miR-147**	27.998
**hsa-miR-25***	2.777	**hsa-miR-335***	6.032	**hsa-miR-92b***	28.949
**hsa-miR-564**	2.805	**hsa-miR-554**	6.091	**hsa-miR-515-5p**	30.258
**hsa-miR-545**	2.807	**hsa-miR-150**	6.103	**hsa-miR-517c**	31.942
**hsa-miR-22***	2.811	**hsa-miR-521**	6.140	**hsa-miR-373***	34.484
**hsa-miR-548d-5p**	2.836	**hsa-miR-615-3p**	6.170	**hsa-miR-524-3p**	34.725
**hsa-miR-891a**	2.840	**hsa-miR-551b***	6.242	**hsa-miR-454***	35.910
**hsa-miR-26a-2***	2.880	**hsa-miR-199a-3p**	6.426	**hsa-miR-133a**	37.907
**hsa-let-7c**	2.932	**hsa-miR-10a***	6.566	**hsa-miR-133b**	38.930
**hsa-miR-200a***	2.943	**hsa-miR-497**	6.572	**hsa-miR-181c***	42.935
**hsa-miR-30c-2***	3.050	**hsa-miR-9**	6.676	**hsa-miR-373**	45.111
**hsa-miR-184**	3.077	**hsa-miR-516b**	6.816	**hsa-miR-518f**	47.556
**hsa-miR-1271**	3.082	**hsa-miR-525-3p**	6.817	**hsa-miR-921**	69.187
**hsa-miR-302a**	3.167	**hsa-miR-541**	7.187	**hsa-miR-548l**	76.180
**hsa-miR-149***	3.175	**hsa-miR-139-5p**	7.456	**hsa-miR-375**	81.495
**hsa-miR-552**	3.302	**hsa-miR-129-3p**	7.465	**hsa-miR-143**	83.063
**hsa-miR-510**	3.344	**hsa-miR-362-5p**	7.488	**hsa-miR-631**	85.097
**hsa-miR-10a**	3.349	**hsa-miR-217**	7.616	**hsa-miR-153**	100.736
**hsa-miR-130b**	3.361	**hsa-miR-888**	7.620	**hsa-miR-105**	131.645
**hsa-miR-96**	3.388	**hsa-miR-301b**	7.815	**hsa-miR-622**	147.739
**hsa-miR-608**	3.404	**hsa-miR-1224-3p**	7.950	**hsa-miR-122**	198.567
**hsa-miR-485-3p**	3.414	**hsa-miR-519a**	8.064	**hsa-miR-147b**	201.357
**hsa-miR-615-5p**	3.421	**hsa-miR-338-3p**	8.159	**hsa-miR-551b**	264.961

**hsa-miR-149**	3.432	**hsa-miR-198**	8.234	**hsa-miRPlus-C1076**	1046.216
**hsa-miR-302c**	3.435	**hsa-miR-610**	8.235		
**hsa-miR-130b***	3.448	**hsa-miR-517a**	8.245		
**hsa-miR-1255b**	3.461	**hsa-miR-10b**	8.885		
