# Supplementary material for: miR-9 Acts as an OncomiR in Prostate Cancer through Multiple Pathways That Drive Tumour Progression and Metastasis
Source: PLoS One. 2016 Jul 22;11(7):e0159601. doi: 10.1371/journal.pone.0159601 (PMC4957825; doi:10.1371/journal.pone.0159601)
Supplement: S3 Table — (PDF) [file pone.0159601.s006.pdf]

miR-9 acts as an OncomiR in prostate cancer through multiple pathways that drive tumour progression and metastasis

S3 Table: miRNAs identified as significantly dysregulated through high-throughput sequencing of prostate cell lines (M12 vs. P69)

Gene Name	Chromosome	logFC	logCPM	LR	PValue	FDR
hsa-miR-675-5p	chr11	10.48098	6.3352868	56.85745	4.69E-14	3.10E-11
hsa-miR-34a-5p	chr1	5.888364	8.7589316	46.10263	1.12E-11	3.71E-09
hsa-miR-370-3p	chr14	-9.48895	8.659433	35.48923	2.56E-09	5.66E-07
hsa-miR-493-3p	chr14	-9.3606	5.9076875	32.8014	1.02E-08	1.35E-06
hsa-miR-127-3p	chr14	-8.33681	8.377626	32.79676	1.02E-08	1.35E-06
hsa-miR-493-5p	chr14	-10.3842	6.955522	32.16034	1.42E-08	1.57E-06
hsa-miR-598-3p	chr8	-8.4122	4.9548096	24.67561	6.78E-07	5.75E-05
hsa-miR-136-3p	chr14	-8.47713	5.0025315	24.63068	6.94E-07	5.75E-05
hsa-miR-654-3p	chr14	-5.64906	7.3934053	22.61502	1.98E-06	0.000146
hsa-miR-375	chr2	3.875028	8.0789744	20.23177	6.86E-06	0.000454
hsa-miR-1250-5p	chr17	5.621295	4.6057246	19.64089	9.34E-06	0.000562
hsa-miR-409-3p	chr14	-5.04411	7.2955072	18.90595	1.37E-05	0.000758
hsa-miR-654-5p	chr14	-7.69118	4.2170658	17.79949	2.45E-05	0.00125
hsa-miR-134-5p	chr14	-4.28	5.7937288	17.55702	2.79E-05	0.00126
hsa-miR-1257	chr20	4.664937	4.5637498	17.51255	2.85E-05	0.00126
hsa-miR-1269a	chr4	4.749274	4.630331	17.24964	3.28E-05	0.001356
hsa-miR-147b	chr15	3.55611	5.7494634	16.33096	5.32E-05	0.002071
hsa-miR-381-3p	chr14	-3.89591	9.838635	15.70913	7.39E-05	0.002717
hsa-miR-590-5p	chr7	7.001886	3.4677385	14.50294	0.00014	0.004876
hsa-miR-411-5p	chr14	-4.10567	7.8055164	14.29948	0.000156	0.005161
hsa-miR-95-3p	chr4	2.77126	6.3715901	14.09168	0.000174	0.005489
hsa-miR-9-5p	chr1, chr5, chr15	3.017321	7.3062746	13.10485	0.000295	0.008658
hsa-miR-136-5p	chr14	-6.58187	3.2398911	13.06526	0.000301	0.008658
hsa-miR-16-2-3p	chr3	-2.9334	11.075447	12.89995	0.000329	0.009064
hsa-miR-548ar-3p	chr13	6.476366	3.09203	12.52451	0.000402	0.010393
hsa-miR-4421	chr1	6.476366	3.09203	12.48961	0.000409	0.010393
hsa-miR-424-3p	chrX	-3.18094	10.243336	12.42388	0.000424	0.010393
hsa-miR-191-3p	chr3	-3.46682	5.4169411	12.23556	0.000469	0.011086
hsa-let-7f-1-3p	chr9	-3.99859	4.5650288	11.95661	0.000545	0.012308
hsa-miR-196a-5p	chr17	2.820411	8.1780643	11.91188	0.000558	0.012308
hsa-miR-656-3p	chr14	-6.42394	3.0954587	11.7599	0.000605	0.012604
hsa-miR-411-3p	chr14	-5.12293	4.2162943	11.74745	0.000609	0.012604
hsa-miR-433-3p	chr14	-6.9885	3.5322098	11.61041	0.000656	0.013156

hsa-miR-382-5p	chr14	-3.94655	5.1898311	11.36491	0.000748	0.014573
hsa-miR-485-3p	chr14	-6.54944	3.1794872	11.16467	0.000834	0.015769
hsa-miR-379-5p	chr14	-3.37206	7.8662884	11.08155	0.000872	0.016033
hsa-miR-301b-3p	chr22	3.474327	4.8391433	10.97718	0.000922	0.016504
hsa-miR-3065-5p	chr17	4.631569	3.6242743	10.86462	0.00098	0.017076
hsa-miR-487b-3p	chr14	-6.00228	2.8186083	10.75552	0.00104	0.017406
hsa-miR-369-3p	chr14	-4.26928	4.2734684	10.68631	0.001079	0.017406
hsa-miR-10a-5p	chr17	2.304609	13.258105	10.6622	0.001093	0.017406
hsa-miR-548ah-3p	chr4	3.349915	4.6115115	10.64398	0.001104	0.017406
hsa-miR-323b-3p	chr14	-4.83247	4.1473728	10.54316	0.001166	0.017954
hsa-miR-3664-5p	chr11	6.013099	2.7836886	10.37589	0.001277	0.019209
hsa-miR-184	chr15	3.087705	4.8152331	10.24206	0.001373	0.020195
hsa-miR-944	chr3	-5.36328	4.555844	10.04392	0.001529	0.021997
hsa-miR-152-3p	chr17	2.315802	9.1330732	9.628353	0.001916	0.02691
hsa-miR-382-3p	chr14	-5.78871	2.6370488	9.594902	0.001951	0.02691
hsa-miR-337-3p	chr14	-5.61139	2.5264609	9.041296	0.002639	0.03566
hsa-miR-576-3p	chr4	2.469907	5.29373	8.997121	0.002704	0.035779
hsa-miR-338-3p	chr17	-5.42373	2.4247609	8.958831	0.002761	0.035779
hsa-miR-199a-3p	chr19	3.081538	4.3007655	8.926641	0.00281	0.035779
hsa-miR-323a-3p	chr14	-3.70565	4.8942093	8.778075	0.003049	0.03808
hsa-miR-98-3p	chr8	-5.41038	2.4082323	8.648416	0.003273	0.04013
hsa-miR-2682-5p	chr1	2.407057	7.2944211	8.566853	0.003423	0.041205
hsa-let-7d-3p	chr9	-3.43439	7.5190379	8.393942	0.003765	0.044505
hsa-miR-122-5p	chr18	-4.44091	3.0241506	8.331172	0.003897	0.04526
hsa-miR-328-3p	chr16	-3.14837	5.5455648	8.291738	0.003983	0.045456
hsa-miR-597-3p	chr8	3.893616	3.3102441	8.251362	0.004072	0.045691
hsa-miR-548ao-3p	chr8	5.492478	2.4685463	8.212879	0.004159	0.045863
hsa-miR-372-3p	chr19	3.865788	3.2594223	8.175138	0.004247	0.045863
hsa-miR-4659a-3p	chr8	5.492478	2.4685463	8.154538	0.004295	0.045863
hsa-miR-3691-5p	chr16	5.492478	2.4685463	8.097273	0.004433	0.046177
hsa-miR-485-5p	chr14	-5.43541	2.4123409	8.084636	0.004464	0.046177
hsa-miR-369-5p	chr14	-3.42558	4.0415502	7.964503	0.00477	0.047314
hsa-miR-196a-3p	chr12	3.483345	3.6823701	7.958197	0.004787	0.047314
hsa-miR-187-3p	chr18	3.824204	3.2629259	7.957606	0.004789	0.047314
hsa-miR-548u	chr6	2.136299	6.0780429	7.863472	0.005044	0.048622
hsa-miR-548o-3p	chr7	2.632128	4.7902015	7.855073	0.005068	0.048622
hsa-miR-377-3p	chr14	-4.07126	3.3063933	7.670454	0.005613	0.052389
hsa-miR-573	chr4	2.533832	4.8411543	7.662799	0.005637	0.052389
hsa-miR-3127-5p	chr2	3.327382	3.5737832	7.643444	0.005698	0.052389
hsa-miR-490-3p	chr7	-5.16585	2.2770809	7.567163	0.005944	0.053257
hsa-miR-579-3p	chr5	3.715198	3.1590142	7.480956	0.006235	0.053257

hsa-miR-495-3p	chr14	-3.57425	4.1461033	7.430418	0.006413	0.053257
hsa-miR-543	chr14	-2.77738	6.8061615	7.419707	0.006451	0.053257
hsa-miR-126-3p	chr9	1.792786	12.074767	7.415971	0.006465	0.053257
hsa-miR-125b-5p	chr11, chr21	-2.11366	12.478931	7.412455	0.006477	0.053257
hsa-miR-127-5p	chr14	-5.20605	2.2852835	7.392657	0.006549	0.053257
hsa-miR-539-3p	chr14	-3.63397	3.7168493	7.387283	0.006569	0.053257
hsa-miR-23a-5p	chr19	-3.00936	4.6082631	7.385263	0.006576	0.053257
hsa-miR-10a-3p	chr17	2.230213	7.2016388	7.379602	0.006597	0.053257
hsa-let-7b-3p	chr9	-2.58209	5.2834221	7.30702	0.006869	0.054319
hsa-miR-296-3p	chr20	-2.67863	7.6420561	7.300799	0.006892	0.054319
hsa-miR-450b-5p	chrX	2.147228	7.6617199	7.251046	0.007086	0.055187
hsa-let-7f-2-3p	chrX	-2.46118	5.4015447	7.197493	0.007301	0.056197
hsa-miR-128-3p	chr2, chr3	-2.03285	11.215315	7.111267	0.00766	0.057844
hsa-miR-148b-3p	chr12	1.784121	10.907865	7.104476	0.007689	0.057844
hsa-miR-329-3p	chr14	-3.57835	2.979897	7.02224	0.00805	0.059505
hsa-miR-125b-2-3p	chr21	3.593308	3.1091984	7.013499	0.00809	0.059505
hsa-let-7a-3p	chr9, chr22	-2.21176	6.9095419	6.878635	0.008723	0.063459
hsa-miR-181a-2-3p	chr9	-2.13046	10.342785	6.852166	0.008853	0.063706
hsa-miR-2682-3p	chr1	3.336262	3.6144046	6.758249	0.009332	0.066424
hsa-miR-3129-3p	chr2	-4.93673	2.1479022	6.696228	0.009662	0.066532
hsa-miR-15b-5p	chr3	1.825168	10.230745	6.669626	0.009807	0.066532
hsa-miR-92a-1-5p	chr13	-2.438	8.2641077	6.654721	0.009889	0.066532
hsa-miR-758-3p	chr14	-2.84456	4.9815014	6.647138	0.009932	0.066532
hsa-miR-6866-5p	chr17	5.139035	2.2762212	6.646976	0.009932	0.066532
hsa-miR-642a-5p	chr19	5.139035	2.2762212	6.643882	0.00995	0.066532
hsa-miR-512-3p	chr19	5.139035	2.2762212	6.613384	0.010122	0.067004
hsa-miR-3116	chr1	5.139035	2.2762212	6.579103	0.010318	0.067631
hsa-miR-3200-3p	chr22	3.353912	2.9303525	6.443731	0.011134	0.072265
hsa-miR-33a-3p	chr22	2.766316	3.9288706	6.364888	0.01164	0.074813
hsa-miR-148a-3p	chr7	1.935925	12.639621	6.345345	0.011769	0.074914
hsa-miR-1185-1-3p	chr14	-2.74844	4.8972821	6.264789	0.012316	0.07765
hsa-miR-431-3p	chr14	-4.96702	2.1520145	6.236076	0.012517	0.078174
hsa-miR-548e-3p	chr10	1.857489	5.7167086	6.206615	0.012727	0.078743
hsa-miR-339-3p	chr7	-2.11746	9.259144	6.137829	0.013232	0.081107
hsa-miR-1910-5p	chr16	-3.04462	3.7401283	6.048531	0.013918	0.084529
hsa-miR-3940-5p	chr19	-5.52255	2.4205882	5.964598	0.014596	0.087295
hsa-miR-3139	chr4	4.923451	2.1681413	5.959638	0.014637	0.087295
hsa-miR-570-3p	chr3	4.923451	2.1681413	5.886894	0.015254	0.090162
hsa-miR-3170	chr13	4.923451	2.1681413	5.866616	0.015431	0.090399
hsa-miR-548k	chr11	1.894143	8.2768119	5.796123	0.016062	0.09327
hsa-miR-3677-3p	chr16	2.475676	4.2133491	5.723648	0.016738	0.096149

hsa-miR-889-3p	chr14	-2.7791	4.9132735	5.712154	0.016848	0.096149
hsa-miR-7-1-3p	chr9	1.764288	6.1247875	5.606257	0.017896	0.10126
hsa-miR-618	chr12	2.138383	4.9344568	5.476391	0.019275	0.107284
hsa-miR-224-5p	chrX	-1.94772	6.9168376	5.475387	0.019286	0.107284
hsa-miR-556-3p	chr1	2.211928	4.6062099	5.460845	0.019447	0.107284
hsa-miR-7-5p	chr9, chr15, chr19	1.743322	9.0592988	5.406258	0.020065	0.109775
hsa-miR-365a-5p	chr16	1.712045	6.8402291	5.371329	0.02047	0.110186
hsa-miR-1185-2-3p	chr14	-5.06289	2.1753721	5.371133	0.020473	0.110186
hsa-miR-643	chr19	3.135802	2.7983221	5.334908	0.020902	0.111592
hsa-miR-520g-3p	chr19	4.669893	2.0502485	5.202568	0.022554	0.119373
hsa-miR-3131	chr2	4.669893	2.0502485	5.166451	0.023027	0.119373
hsa-miR-597-5p	chr8	3.100262	2.8020182	5.16341	0.023068	0.119373
hsa-miR-195-5p	chr17	4.669893	2.0502485	5.159725	0.023117	0.119373
hsa-miR-9-3p	chr1, chr5, chr15	2.620518	3.5080849	5.148871	0.023261	0.119373
hsa-miR-34c-5p	chr11	-1.9367	5.545866	5.093011	0.024022	0.122091
hsa-miR-363-3p	chrX	-1.79728	6.3615813	5.083104	0.02416	0.122091
hsa-miR-374a-3p	chrX	1.696491	8.7016884	4.976214	0.025698	0.12888
hsa-miR-376c-3p	chr14	-2.75651	3.0566237	4.842516	0.027766	0.138205
hsa-miR-1303	chr5	2.000339	4.6920058	4.748333	0.029327	0.144883
hsa-miR-449c-5p	chr5	2.112273	4.3874344	4.731084	0.029622	0.145259
hsa-miR-299-3p	chr14	-2.73452	4.0777917	4.712507	0.029944	0.14528
hsa-miR-503-3p	chrX	-2.59151	3.727045	4.705543	0.030066	0.14528
hsa-miR-409-5p	chr14	-2.77587	3.0606133	4.605431	0.031871	0.152078
hsa-miR-30b-5p	chr8	1.513539	10.100684	4.602152	0.031932	0.152078
hsa-miR-1278	chr1	1.677183	7.1805861	4.587985	0.032197	0.152245
hsa-miR-580-3p	chr5	2.49057	3.4251131	4.5718	0.032502	0.1526
hsa-miR-1293	chr12	-2.64721	3.4468847	4.517648	0.033547	0.156395
hsa-miR-6511b-3p	chr16	-4.24142	1.8420774	4.436395	0.03518	0.159676
hsa-miR-148b-5p	chr12	1.663716	7.2717588	4.431588	0.03528	0.159676
hsa-miR-1268b	chr17	4.362065	1.920639	4.422736	0.035463	0.159676
hsa-miR-5000-3p	chr2	4.362065	1.920639	4.422736	0.035463	0.159676
hsa-miR-3136-5p	chr3	4.362065	1.920639	4.422736	0.035463	0.159676
hsa-miR-1294	chr5	-2.43317	3.6137166	4.411475	0.035698	0.159676
hsa-miR-4517	chr16	-4.21201	1.8338735	4.3789	0.036386	0.161663
hsa-miR-224-3p	chrX	-2.05186	4.832077	4.353778	0.036927	0.162147
hsa-miR-146a-3p	chr5	-4.19767	1.8297875	4.351093	0.036985	0.162147
hsa-miR-340-3p	chr5	1.569211	5.7601478	4.330719	0.03743	0.163019
hsa-miR-138-5p	chr3, chr16	-2.13388	6.5033582	4.267339	0.038852	0.168104
hsa-miR-26a-2-3p	chr12	-2.21646	4.1429851	4.207465	0.040246	0.173007
hsa-miR-590-3p	chr7	1.563978	7.111982	4.189975	0.040664	0.173673

hsa-miR-3935	chr16	-2.81498	2.4975099	4.151213	0.041605	0.176553
hsa-miR-151a-3p	chr8	1.430195	10.844449	4.116336	0.042471	0.178699
hsa-miR-138-1-3p	chr3	-2.79921	3.9424093	4.10921	0.04265	0.178699
hsa-miR-1272	chr15	2.478951	3.0055759	4.038241	0.04448	0.185194
hsa-miR-137	chr1	1.655684	6.9304506	3.971105	0.046287	0.191514
hsa-miR-374b-3p	chrX	1.506554	7.4790533	3.876637	0.048963	0.201324
