# Supplementary material for: miR-9 Acts as an OncomiR in Prostate Cancer through Multiple Pathways That Drive Tumour Progression and Metastasis
Source: PLoS One. 2016 Jul 22;11(7):e0159601. doi: 10.1371/journal.pone.0159601 (PMC4957825; doi:10.1371/journal.pone.0159601)
Supplement: S4 Table — (PDF) [file pone.0159601.s007.pdf]

S4 Table: Moribundity scale for *in-vivo* experiments using mice

Parameter	0	1	2
**General Appearance**<br>Dehydration, decreased body weight <10%, abnormal posture, swelling, prolapses			
**Skin/Fur**<br>Discoloration, urine stain, pallor, redness, cyanosis, wounds, sores, abscess, ulcer, ruffled fur			
**Eyes**<br>Exophthalmos, microphthalmis, ptosis, reddened eye, lacrimation, opacity			
**Locomotion**<br>Hyperactivity, lethargy, coma, ataxia, tremors			
**Nose, Mouth, Head**<br>Tilted head, nasal discharge, malocclusion, salivation			
**Respiration**<br>Sneezing, dyspnea, tachypnea, rales			
**Urine**<br>Hematuria, poly/anuria			
**Feces**<br>Diarrhea, hematochezia			
